# Adaptive Evolution in TRIF Leads to Discordance between Human and Mouse Innate Immune Signaling

**DOI:** 10.1093/gbe/evab268

**Published:** 2021-12-06

**Authors:** Edel M Hyland, Andrew E Webb, Kathy F Kennedy, Z Nevin Gerek Ince, Christine E Loscher, Mary J O’Connell

**Affiliations:** 1 Bioinformatics and Molecular Evolution Group, School of Biotechnology, Dublin City University, Glasnevin, Dublin 9, Ireland; 2 School of Biological Sciences, Queen’s University Belfast, Belfast, United Kingdom; 3 Immunomodulation Group, School of Biotechnology, Dublin City University, Glasnevin, Dublin 9, Ireland; 4 Institute for Genomics and Evolutionary Medicine, Temple University, Philadelphia, Pennsylvania, USA; 5 Computational and Molecular Evolutionary Biology Group, School of Life Sciences, Faculty of Medicine and Health Sciences, University of Nottingham, Nottingham, United Kingdom

**Keywords:** innate immune signaling, positive selection, ancestral gene resurrection

## Abstract

The TIR domain-containing adapter inducing IFN-β (TRIF) protein is an innate immune system protein that mediates the MyD88-independent toll-like receptor response pathway in mice and humans. Previously, we identified positive selection at seven distinct residues in mouse TRIF (mTRIF), as compared with human and other mammalian orthologs, thus predicting protein functional shift in mTRIF. We reconstructed TRIF for the most recent common ancestor of mouse and human, and mutated this at the seven sites to their extant mouse/human states. We overexpressed these TRIF mutants in immortalized human and mouse cell lines and monitored TRIF-dependent cytokine production and gene expression induction. We show that optimal TRIF function in human and mouse is dependent on the identity of the positively selected sites. These data provide us with molecular data relating observed differences in response between mouse and human MyD88-independent signaling in the innate immune system with protein functional change.

Significance

There are many similarities between human and mouse immune systems. However, there are a number of cases, for example, TRIF-dependent responses, where the human and mouse respond differently to infection. Previously, we proposed that species-specific positive selection in immune system genes may be a predictor of species-specific immune response (Webb et al. 2015). Here we perform immunological assays and demonstrate that sites under positive selection in the mouse *TRIF* (*mTRIF*) gene drive the known mouse-specific response. These findings show that the observed phenotypic/physiological differences between human and mouse in the MyD88-independent pathway response are driven by sites under positive selection in *mTRIF*.

## Introduction

There are numerous cases whereby the mouse model fails to fully mimic human physiology (Sango et al. 1995; Rangarajan and Weinberg 2003; Hatzipetros et al. 2014), including differences in the immune response (Sellers 2017). Understanding the molecular underpinnings of such variation between human and mouse will improve experimental design and relevance of mouse models. Given that mouse and human lineages diverged from a common ancestor ∼88 Ma (Hedges et al. 2006), their life histories and evolutionary trajectories in the intervening ∼180 Myr have given rise to lineage-specific mutations. By identifying genes (and sites) under species-specific positive selection using comparative phylogenomics we can approximate functional divergence between human and mouse. Indeed we, and others have shown that sites under positive selection are involved in protein functional shift for a variety of proteins (Goriely et al. 2005; Levasseur et al. 2006; Loughran et al. 2012). Therefore, comparative phylogenomics can be used to systematically identify functionally discordant pathways between mouse and human, to assess the utility of mice more accurately as models for human biology and disease.

Variation in immune response between human and mouse is not unsurprising given that idiosyncratic diets, climates, and exposure to distinct pathogens shapes immune responses (Mestas and Hughes 2004; Kammerer et al. 2007; Barreiro et al. 2010; Vasseur and Quintana-Murci 2013). Indeed, genes involved in the innate immune response have been identified as some of the most rapidly evolving across a broad range of taxa (Obbard et al. 2009; McTaggart et al. 2012; Lazzaro and Clarke 2013; Behrman et al. 2018; Adrian et al. 2019), amplifying differences between species. Previously, we identified positive selection in 35 innate immune system genes in mouse (Webb et al. 2015) and our aim in this study is to determine the functional consequence(s) of positive selection in one of these genes. We chose to focus on the mouse TRIF (mTRIF) protein as previous studies demonstrated divergence in TRIF-dependent signaling between mouse and human (see below). Furthermore, the number of predicted residues in *mTRIF* that contribute to the signature of positive selection are amenable to experimentation, and the assays to monitor TRIF function are well established.

**Fig. 1.**
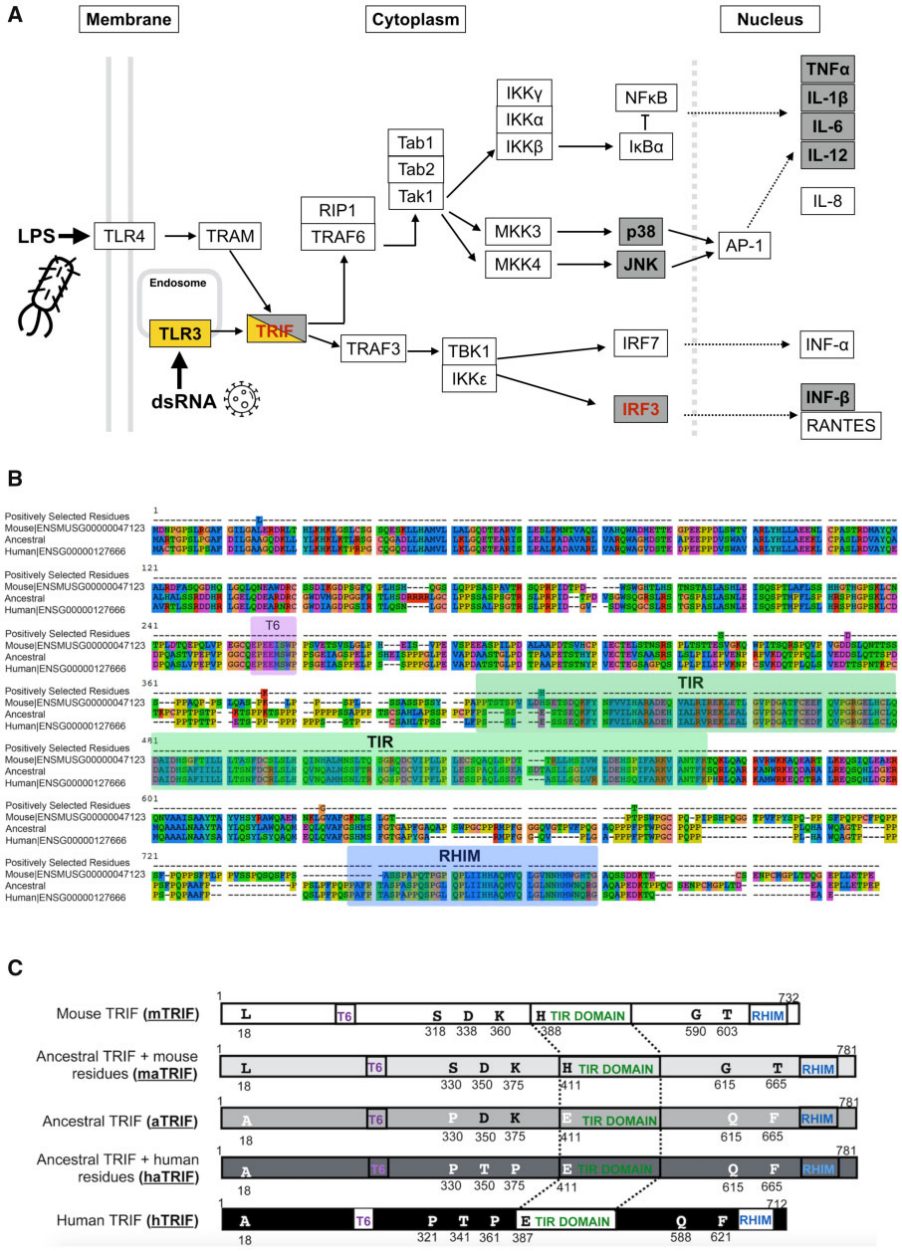
Signatures of adaptive evolution identified in *TRIF* and TRIF-dependent signaling pathways. (*A*) Schematic adapted from the Kyoto Encyclopedia of Genes and Genomes (KEGG) illustrating TRIF-dependent pathways in toll-like signaling in innate immunity. Selective pressure variation analysis was performed for both lineage-specific (model A) and site-specific (model 8) selection. Genes boxed in gray represent those that are predicted to have signatures of positive selection; gene names in bolded black contain residues that are predicted to be under site-specific selection, and genes in red are genes under selection in the mouse lineage only. Highlighted in yellow are *TLR3* and *TRIF* predicted to be under selection in mouse by our earlier analysis (Webb et al. 2015). (*B*) A portion of the PRANK amino acid alignment of TRIF homologues showing the human, mouse, and ancestral TRIF protein sequences. The positions of the positively selected sites are indicated, as well as the position (relative to human—O'Neill and Bowie 2007) of the three known functional TRIF domains: Traf6 binding motif (T6), Toll/interleukin-1 receptor (TIR) domain, and the receptor-interacting protein (RIP) homotypic interaction motif (RHIM). (*C*) Schematic illustrating the different protein sequences of TRIF used in this study. Residues highlighted in the mouse protein (mTRIF) are those predicted to be under positive selection. Their position relative to the three functional domains is indicated. The position and identity of the homologous residues in the hTRIF, the reconstructed ancestral TRIF (aTRIF), the humanized ancestral TRIF protein (haTRIF), and the murinized ancestral TRIF (maTRIF) are also shown.

TRIF is an intracellular adapter protein that acts in the MyD88-independent toll-like receptor (TLR) pathway, signaling specifically through TLR3 and TLR4 (reviewed Ullah et al. 2016) (fig. 1*A*). TLR3 and TLR4 receptors are activated by viral (dsRNA) and bacterial (lipopolysaccharide [LPS]) signals respectively, promoting the antiviral and proinflammatory response. Specifically, TRIF is recruited to activated TLR3 and TLR4, and through its interaction with the signaling proteins RIP1 and TRAF6 leads to the downstream activation of IRF3 transcription factor. This induces the expression of type-1 interferons and pro-inflammatory cytokines such as RANTES, and numerous interleukins namely, IL6, IL8, IL12, and IL-1β (fig. 1*A*).

Documented inconsistencies exist in TLR3 and TLR4 signaling between mouse and human. First, the structure of the *TLR3* and *TLR4* genes themselves differ between mouse and human (Rehli 2002, Iwami et al. 2000), and well-documented differences exist in the regulation of TLR3 expression between both species (Heinz et al. 2003). Second, the loss of TLR3 function leads to discordant phenotypes in mice and humans. For example, TLR3-deficient mice show disruption in susceptibility to many viruses ranging from increased susceptibility to increased resistance phenotypes depending on the virus, indicating a broad role for TLR3 signaling in protection against viral infections in mice (reviewed Zhang et al. 2013). In humans however, individuals born with deficiencies in TRL3 signaling show increased susceptibility to only a single virus, HSV-1 as TLR-3-independent signaling contributes more substantially to antiviral immunity in humans (Zhang et al. 2013). Furthermore, differences exist in the regulation of TRIF-dependent cytokine production between mouse and human involving PKC-α (Langlet et al. 2010), and TLR3-specific activation leads to discordant responses between rodents and primates (Mitchell et al. 2014). Taken together, this evidence suggests that TRIF-dependent pathways and signaling in mouse and human have functionally diverged.

## Results

### The MyD88-Independent Signaling Pathway Shows Evidence of Divergence between Human and Mouse

In a previous study, we reported that the sequences of 37 innate immune response genes, including *mTRIF*, showed differences in selective pressure between human and mouse orthologs (Webb et al. 2015). Here, we extended this selective pressure analysis using CodeML (Yang 2007) to include 11 additional genes involved in TRIF-dependent signaling and increased our sequence data set from 21 to 43 mammalian genomes (supplementary tables S1 and S2, Supplementary Material online). Again, we detected multiple differences in selective pressures between mouse and human for 9 TRIF-related proteins (fig. 1*A* and table 1). We reconfirmed lineage-specific positive selection in *TRIF* in the mouse lineage (Webb et al. 2015), although there was not a complete overlap in the identity of the amino acids under selection between both studies (supplementary fig. S3*A*, Supplementary Material online). This presumably is due to the additional genomes present in our analysis resulting in changes in the multiple sequence alignment. For the *TRIF* alignment, we determined the confidence score to be 0.883, and <4% of the columns in this alignment were deemed “unreliable.” We also detected mouse lineage-specific positive selection in *IRF3*, the major downstream transcription factor in the MyD88-independent signaling cascade. Interestingly, none of the genes analyzed are predicted to be under positive selection in the human lineage. These results further suggest that orthologous genes in the TRIF-dependent immune response pathways have functionally diverged between human and mouse. Furthermore, we identified site-specific positive selection downstream in the same pathway in the following genes: p38, and the cytokines TNFα, IL-1B, IL-6, IL-12A, and Interferon β1. Overall, these findings indicate a strong selective pressure for change of function in the MyD88-independent signaling cascade.

**Table 1.** Summary of CodeML-Positive Selection Analysis on TRIF-Related Immune Response Genes

Gene Name	Type of Positive Selection Predicted^a^	Sites^b^	*P* Value of Positively Selected Sites^c^
TRIF	Lineage specific, mouse	18, 218, 270, 318, 338, 360, 375, 387, 388, 512, 603	11 > 0.50, 3 > 0.90, 0 > 0.99
	Site specific	180, 332, 362, 369, 406, 424, 425, 465, 479, 496	10 > 0.50, 4 > 0.90, 0 > 0.99
TLR3	Lineage specific, mouse	266, 297, 603	3 > 0.50, 0 > 0.90, 0 > 0.99
IRF3	Lineage specific, mouse	96, 140, 152, 229	4 > 0.50, 2 > 0.90, 0 > 0.99
p38-β/MAPK11	Site specific	**16**	1 > 0.50, 1 > 0.90, **1 > 0.99**
TNFα	Site specific	16, **71**, 74, 75, 163, 164, 173, 184, **186**, 194, 249	11 > 0.50, 5 > 0.90, **2 > 0.99**
IL-1B	Site specific	109, 143, 245, 246, 247, 252, 281, 284, 285, 289, 291, 308, 332, 360, 400, 445, 456	17 > 0.50, 2 > 0.90, 0 > 0.99
IL-6	Site specific	102, 128, 149, 156, 164, 166, 173, 176, 187, 231, 250, 254, 255, 258, 259, 262, 273, 286, 287, 288, 289, 292, 298, 299, 320, 324, 328, 333, 335	29 > 0.50, 4 > 0.90, 0 > 0.99
IL-12A	Site specific	21, 110, 152, 178, 190, 277, 281, 327	8 > 0.50, 2 > 0.90, 0 > 0.99
INF β1	Site specific	25, 26, 28, 36, 37, **43,** 48, 50, 52, 78, 82, 90, 111, 114, 119, 122,135, 137, 138, 140, 145, 153, 156, 157, 173, 188, 191	27 > 0.50, 8 > 0.90, **1 > 0.99**

a For site-specific selection, results are based on model M8 alone (reporting BEB sites).

b For genes under site-specific selection, site numbers refer to position in the multiple sequence alignment used for CodeML analysis. For lineage-specific selection, site number (in red) refers to the amino acid position in lineage/species where selection has been detected.

c The associated posterior probability is indicated through formating: underlined >0.90, in bold >0.99, and no formatting implies 0.50 < PP < 0.90.

### A Resurrected Mouse–Human Ancestral TRIF Protein Shows Species-Specific Functionality

Mammalian *TRIF* contains three annotated domains: highly conserved TIR and TRAF6 binding domains, and a less well-conserved RHIM (receptor-interacting-protein homotypic interaction motif) (fig. 1*B*). Of the 10 sites predicted to be under selection in *mTRIF*, we chose the seven with the highest confidence to focus on (see supplementary fig. S3*B*, Supplementary Material online for details). Of those seven amino acid residues under positive selection, only one is located within an annotated (TIR) domain (fig. 1*B*). To understand the evolutionary relevance of the positively selected mouse amino acids, we employed ancestral gene reconstruction (AGR) (for review, see Thornton 2004). Using FastML (Ashkenazy et al. 2012), we reconstructed the most recent common ancestor of mouse–human TRIF (*aTRIF*) (as in Bickelmann et al. 2015, Voordeckers et al. 2012, Randall et al. 2016, Castro-Fernandez et al. 2017, and Furukawa et al. 2020), from an alignment of 10 rodent and mammalian genomes. This alignment obtained a Guidence2 confidence score of 0.947, with unreliable columns making up <1% of the total alignment. This predicted aTRIF sequence was 781 amino acids in length, with a median posterior probability score of 0.934 for individual codons (supplementary fig. S4, Supplementary Material online), giving us sufficient confidence in its composition. Interestingly, within the known annotated domains, the predicted aTRIF protein is more similar in sequence to the extant human TRIF (mTRIF) than mTRIF (supplementary table S4, Supplementary Material online). Using gene synthesis, we resurrected this *aTRIF* gene sequence and cloned it into a mammalian overexpression plasmid under the control of the constitutive CMV promoter.

**Fig. 2.**
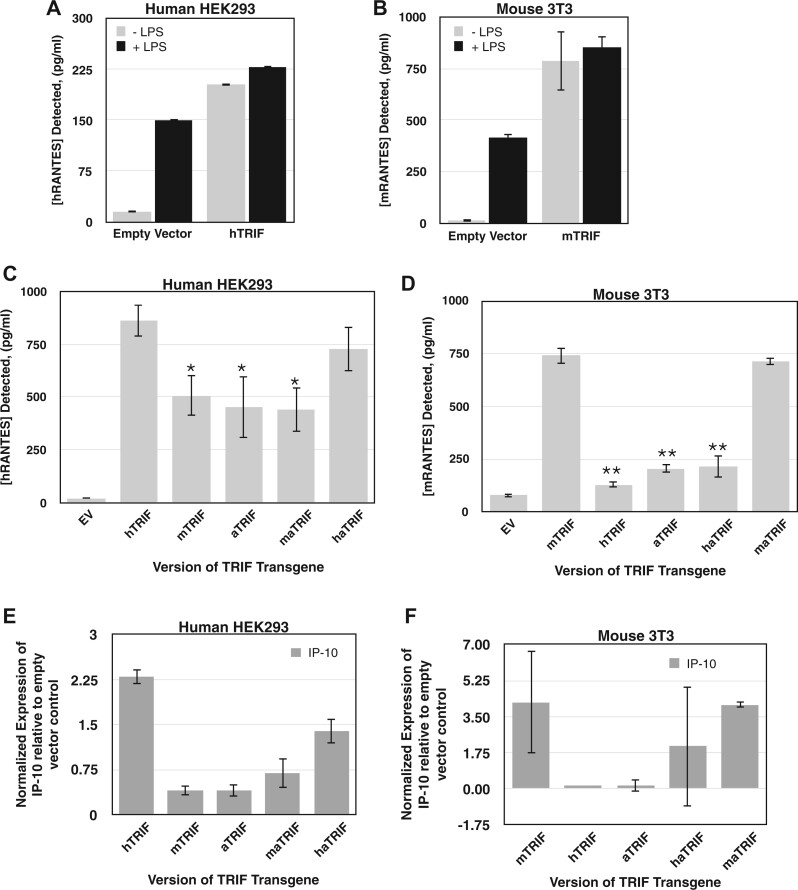
Amino acid residues under positive selection in *TRIF*, modulate species-specific function. Functional analysis of *TRIF* gene constructs. TRIF-induced RANTES production for human HEK 293 cells (*A* + *C*) and mouse 3T3 cells (*B* + *D*). Cell lines were transiently transfected with plasmids constitutively expressing each of the versions of the *TRIF* transgenes as indicated, or an empty vector (EV) control. In (*A*) and (*B*), cells were stimulated with LPS 12 h post-infection. At 24 h post transection, supernatants were recovered and assayed for the presence of the RANTES cytokine by ELISA. Each graph represents data from three biological replicates assayed in triplicate from a single day’s experiment. Similar trends were observed on different days. Error bars denote SD. *P < 0.05 compared with hRANTES value in HEK cells. **P < 0.01 compared with mRANTES value in 3T3 cells. Quantitative RT-PCR analysis as outlined in figure indicating the expression of IP-10 in human HEK 293 TLR4 cells (*E*) and in murine 3T3 cells (*F*) expressing the indicated TRIF constructs. Baseline of 0 is set to the expression of IP-10 in cells transfected with the empty vector control.

Initially we sought to verify the functionality of the aTRIF protein and transfected both human embryonic kidney (HEK) 293 TLR4+ cells, and murine fibroblast NIH 3T3 cells with our synthetic gene construct. Additionally, we used two control plasmids that expressed either *hTRIF* or *mTRIF* genes under the same regulation. Upon transfection of the cell lines with each TRIF construct, we used ELISA to monitor the production of the cytokine RANTES (downstream of MyD88-independent TLR4 signaling) as a proxy for TRIF function. The overexpression of hTRIF and mTRIF in their native context, HEK 293 cells and 3T3 cells respectively, induces the production of RANTES, bypassing the requirement for LPS, the canonical stimulus for TLR4 signaling (fig. 2*A* and *B*). However, not surprisingly, the expression of hTRIF and mTRIF constructs in their nonnative context, that is, 3T3 cells and HEK 293 cells respectively, significantly decreased the levels of RANTES produced compared with their native hosts, indicating reduced TLR4 signaling by these cross-species TRIF proteins. For the ancestral aTRIF construct, RANTES production was stimulated to levels above those detected for the empty vector control for both cell types indicating that the aTRIF protein is functional in this assay. However, the signal obtained was greater in HEK293 cells suggesting that aTRIF functions better within the human cellular context (fig. 2*C* and *D*). As a second functional test of each TRIF construct, we assessed the expression of the chemokine IP-10 upon transfection of both HEK 293 and 3T3 cells using RT-qPCR. IP-10 gene expression occurs in response to LPS stimulation of TLR4 and is TRIF dependent (Fitzgerald et al. 2003b). Our expression data corroborate the ELISA results showing that in HEK293 cells, TLR4 signaling is supported by hTRIF and to a much lesser extent mTRIF and aTRIF (fig. 2*E*). In murine NIH 3T3 cells robust IP-10 expression is detected for mTRIF, but negligible IP-10 RNA is present when hTRIF or aTRIF is present in the cells (fig. 2*F*).

### Amino Acid Sites Predicted to Be under Positive Selection Confer Optimal TRIF Function in Native Cellular Context

The resurrected *aTRIF* sequence provided us with the appropriate background to test the effects of species-specific mutations on functional diversification in *TRIF* (Harms and Thornton 2010). An alignment of the *aTRIF* sequence with *mTRIF* and *hTRIF* (fig. 1*B*) identifies amino acids in *aTRIF* that are homologous to the seven sites under positive selection in mouse. Most of these sites (five out of seven) reflect the specific residue found in extant *hTRIF*, which is expected given our prediction that positive selection has acted specifically on the mouse, and not human lineage. The fact that two of the mouse sites under selection, Asp350 and Lys360, are present in the predicted ancestral sequence, will be discussed below. We hypothesize that the seven sites under positive selection in *mTRIF* confer an altered function to mTRIF-signaling that will be absent in the aTRIF protein. Furthermore, by combining AGR with rational mutagenesis (Loughran et al. 2012; Baldwin et al. 2014), we can approximate the evolution of functional divergence in TRIF protein along the human and mouse lineages. We generated two “pseudo-evolved” versions of aTRIF: 1) mouse ancestral TRIF (*maTRIF*) and 2) human ancestral TRIF (*haTRIF*) where the homologous sites in *aTRIF* were mutated to reflect those found in either present day *mTRIF* or *hTRIF*, respectively (fig. 1*C*). Again, we used gene synthesis to resurrect these different versions of aTRIF and determined the effects of the mutations in the protein background in which the substitutions most probably occurred.

Analysis of the different versions of the ancestral TRIF protein indicated that in HEK 293 cells, maTRIF led to impaired signaling, whereas the haTRIF construct restored the ability of aTRIF to produce RANTES at a level almost comparable with hTRIF (fig. 2*C*). In contrast in murine 3T3 cells, the haTRIF protein significantly compromised the production of RANTES, whereas the maTRIF protein restored signaling through the pathway to wild-type mTRIF levels (fig. 2*D*). Again, quantitative RT-PCR analysis of IP-10 mRNA supported our ELISA data and showed that in HEK293 human cells, haTRIF significantly (*P* value > 0.05) increased IP-10 mRNA abundance over aTRIF, whereas the mouse-specific maTRIF did not (fig. 2*E*). Conversely, the maTRIF restored IP-10 mRNA expression levels to that of mTRIF levels in 3T3 cells, indicating that the mouse-specific substitutions were sufficient to improve the functionality of the ancestral *TRIF* gene to normal levels in this species. However, the haTRIF construct did not robustly alter IP-10 expression levels in the mouse cellular context (fig. 2*F*).

To determine whether expression differences between our TRIF constructs could account for the observed phenotypic differences, we determined the levels of TRIF mRNA using RT-qPCR with construct-specific primers (supplementary fig. S5, Supplementary Material online). Our data indicate strong expression (>15 times relative to empty vector control) of each *TRIF* gene from the CMV promoter for all constructs in HEK 293 cells, except *maTRIF*. In 3T3 cells, although RNA abundance of each construct was lower than in HEK 293 cells, reproducible levels were detected for all, but the maTRIF construct. That said, there is clearly some variability of expression between the constructs in different cellular context. For 3T3 cells however, the pattern of expression variability is not consistent with the phenotypic results. For example, the maTRIF construct is the least expressed gene but shows optimal TRIF signaling in both the Rantes ELISA and IP-10 expression analysis. Similarly, in HEK cells the ancestral TRIF (*aTRIF*) sequence is the most highly expressed construct yet performs poorly in the functional analysis. Together, these observations are consistent with a lack of obvious correlation between TRIF mRNA abundance and TRIF function in our experimental setup, providing us with confidence in our conclusions about TRIF genotype/phenotype relationship.

### In Silico Analysis of TRIF Protein Structure Predicts That Amino Acids under Positive Selection in Mouse Are Structurally Important

**Fig. 3.**
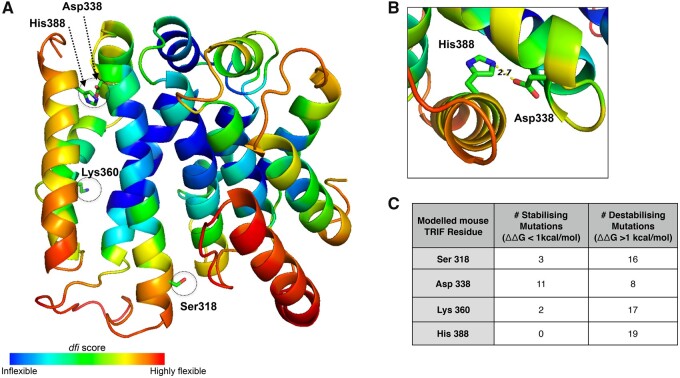
In silico analysis highlights the structural importance of sites under positive selection in mTRIF. (*A*) The predicted structure of a portion of the mTRIF protein (residues Pro110 to Gln394) generated using MODELLER (Fiser and Sali 2003). Residues are highlighted based on their dynamic flexibility index, (dfi) score, as per the indicated scale, and the position of four mouse residues under positive selection are indicated. (*B*) A close up of His 388 and Asp 338, two mTRIF residues under positive selection, in the predicted TRIF protein structure. The shortest distance between their two side chains is indicated (2.7 Å) and suggests a potential interaction between these two residues. Both (*A*) and (*B*) were generated using pymol (Delano 2002). (*C*) A summary of the in silico mutagenesis analysis done on mTRIF protein structure using dezyme software (https://soft.dezyme.com). The number of mutations that led to either a decrease (stabilizing) or increase (destabilizing) in the folding free energy (△△G) of mTRIF is indicated.

Our experimental data lend support to our computational predictions about the altered function of mTRIF. We show that the predicted seven amino acid residues under positive selection in mTRIF; Leu18, Ser318, Asp338, Lys360, His388, Gly590, and Thr603, are required for optimal TRIF-dependent signaling in mouse cells. Clearly a detailed dissection of the contribution of each of the seven residues to mTRIF function is needed. However, the prediction that two of these mouse-specific residues (Asp338 and Lys360) existed in ancestral TRIF, suggests that they are either false positives of our CodeML analysis and are not under positive selection, or they reflect the limitations of AGR (Thornton 2004). In support of this, the haTRIF protein that lacks Asp338 and Lys360 functions equally poorly in mouse cells as the aTRIF protein which retains them, indicating that by themselves, Asp338 and Lys360 do not contribute to TRIF function in mouse in the context of the ancestral protein. Furthermore, both these residues lie in positions of the multiple sequence alignment with low confidence scores, 0.290 for Asp338, and 0.208 for Lys360. However, it is also possible that they are evidence of epistasis between the sites under positive selection (O'Maille et al. 2008; Phillips 2008), whereby Asp338 and Lys360 are required in the context of the mouse sequence to promote the altered function conferred by the remaining five sites, or indeed the identity of the residues has switched back and forth over evolution to account for amino acid substitutions at other positions. In support of epistasis, we used MODELLER (Fiser and Sali 2003) to predict the structure of a portion of mTRIF protein (Supplementary Material online) and identified a possible electrostatic interaction between Asp338 and a second site under positive selection, His388 (fig. 3).

We used our predicted mTRIF protein structure to assess a structure/function role for other mouse sites under positive selection and calculated the dynamic flexibility index (dfi) at each site. A low dfi score signifies high structural conservation and an intolerance to substitutions at that position. A positive correlation exists between dfi score and evolutionary rate (Nevin Gerek et al. 2013). Both Ser318 and His388 have relatively intermediate dfi scores (0.71 and 0.65, respectively) indicating moderate flexibility at these positions (fig. 3*A*). However, in silico mutagenesis, using dezyme PoPMuSiC analysis (Dehouck et al. 2009) (https://soft.dezyme.com) predicts that the majority of substitutions at these sites, including those found in hTRIF, are destabilizing (fig. 3*C*), highlighting their structural importance in mTRIF.

In human cells, we found that the impairment of signaling by the ancestral protein aTRIF is partially restored by the introduction of two specific residues, corresponding to Thr341 and Pro361 in the hTRIF protein, indicating that in the context of the ancestral protein the identity of these residues is important for TRIF signaling in human cells. Molecular dynamic simulations using a predicted hTRIF protein structure (supplementary fig. S6, Supplementary Material online) indicates that for Thr341, the majority of substitutions (14) are destabilizing, whereas replacing Pro361 with all amino acids except glycine, leads to a more stable hTRIF protein. These predicted contrasting effects of mutagenesis highlights the complexity in understanding the molecular basis of positive selection and underscores the need for independent experimental analysis of each of these residues.

## Discussion

This study sought evidence that residues under positive selection in *mTRIF* confer species-specific functionality to TRIF signaling pathway. Our data support this prediction and demonstrate the effectiveness of combining AGR with rational mutagenesis to experimentally test this. That said, we fully acknowledge that there are documented limitations with AGR (Thornton 2004; Ekman et al. 2008) not least that the ancestral *TRIF* sequence presented here is an approximation. To this end, we report high confidence (mean posterior probability per codon = 0.934) in our predicted ancestral sequence, as well as in the identity of the homologous sites under selection. However, we acknowledge that a more robust strategy might be to resurrect multiple versions of aTRIF containing alternative states at the most ambiguously reconstructed positions. If these different versions of aTRIF behaved the same, it would eliminate doubt regarding ancestral state inference and provide a level of robustness to our experimental conclusions.

Furthermore, we acknowledge that placing an ancient protein inside a modern cell and assaying function holds many caveats, not least the possible loss of interaction(s) with vital proteins, and therefore the decreased activity of aTRIF in human and mouse cells is not unexpected. To date, TRIF protein has three known interacting partners: TIR domains of TLRs, RIP, and Traf6. The TRIF sequences mediating these interactions are highly conserved; in fact, previous reports show that the TRIF TIR domain is under purifying selection (Fornarino et al. 2011). As such, the ancestral TRIF protein maintains a high level (79–100%) of sequence similarity with the extant mouse and human proteins at these interaction sites (supplementary table S4, Supplementary Material online). Therefore, it is possible that the known interactions of TRIF are sufficiently supported by the ancient protein in the modern cell. Regardless, our results indicate that despite these limitations to AGR, the activity of aTRIF was recovered in mouse cells by reintroducing the seven amino acids under positive selection found in present day mTRIF.

Given that six of the seven sites under positive selection in *mTRIF* are located in un-annotated regions, their specific (and individual) mechanistic contribution(s) to TRIF function will require further analyses. It is possible that these residues influence TRIFs known functions, for example by modifying its interaction with pathway components. However, given that positive selection is thought to result in protein functional shift (Loughran et al. 2012; Baldwin et al. 2014) it is feasible that all/some of these sites in TRIF mediate a novel interaction/function in mouse. To this end, we acknowledge that our study falls short of uncovering what this might be, and that substantially more in-depth mechanistic analyses need to be undertaken. That said, our experiments have identified a small set of residues in the ancestral TRIF protein that by changing them to be mouse specific or human specific is sufficient to recapitulate TRIF signaling activity within mouse and human cells respectively.

## Conclusion

To conclude, there are many similarities between human and mouse immune systems. However, there are cases where the human and mouse respond differently to infection, for example, TRIF-dependent responses. Previously, we proposed that species-specific positive selection in immune system genes may be a predictor of species-specific immune response (Webb et al. 2015). Here we performed immunological assays and demonstrate that sites under positive selection in the *mTRIF* gene drive the known mouse-specific response. These findings show that the observed phenotypic/physiological differences between human and mouse in the MyD88-independent pathway response are driven by sites under positive selection in *mTRIF*. Alongside the evolutionary implications, this work contributes to efforts in humanizing mouse responses when modeling disease and paves the way for future work on uncovering the species-specific function(s) of mTRIF.

## Materials and Methods

### Selective Pressure Analysis

Selective pressure analyses were performed using codeML from the PAML software package (v4.4) (Yang 2007). Briefly, CodeML examines nested codon-based models of evolution in a maximum-likelihood framework to determine the ratio of nonsynonymous substitutions per nonsynonymous site (Dn) to synonymous substitutions per synonymous site (Ds) (ω = Dn/Ds). We employed both site-specific and branch-specific models to test for positive selection at sites throughout our data set as well as in mouse and human lineages, respectively (Yang 2007). If positive selection is inferred, the posterior probability of the positively selected site is estimated using two calculations: Naïve empirical Bayes (NEB) or Bayes Empirical Bayes (BEB) (Yang 2007). If both BEB and NEB are predicted, we utilized the BEB results as they have been reported to be more robust (Yang 2007). We analyzed 12 genes in the TRIF-dependent immune response pathway (supplementary table S1, Supplementary Material online). Single gene orthologs were identified from the transcripts of 43 high quality genomes (supplementary table S2, Supplementary Material online) using the ENSEMBL Biomart database (https://www.ensembl.org/biomart). This analysis was therefore more in depth than our previous one which utilized up to 21 species (Webb et al. 2015). Alignments for CodeML analysis contained between 28 and 37 species depending on what was available on ENSEMBL (supplementary table S1, and Supplementary Material online). Alignment files can be found in the Supplementary Material online, and the TRIF alignment is shown in supplementary figure S1, Supplementary Material online. Alignment confidence was assessed using Guidance2 (Penn et al. 2010) and confidence scores obtained. Any positively selected sites inferred using CodeML under a given model were mapped back to the species-specific Uniprot protein sequence using software described in Webb et al. (2017).

### Ancestral TRIF Gene Resurrection

The most probable primate-rodent ancestral state for all sites in TRIF was determined using the FastML web-server (Ashkenazy et al. 2012), employing maximum-likelihood methods and the Yang codon model of protein evolution (Yang and Nielsen 2002). Briefly, homologous TRIF sequences from the primates, Human (ENSG00000127666), Marmoset (ENSCJAG00000017459), Gorilla (ENSGGOG00000006675), Macaque (ENSMMUG00000014163), Gibbon (ENSNLEG00000013388), Bushbaby (ENSOGAG00000024483), Chimpanzee (ENSPTRG00000010322), and Orangutan (ENSPPYG00000009418) as well as the rodents Mouse (ENSMUSG00000047123), and Guinea Pig (ENSCPOG00000006486) were aligned using PRANK (Loytynoja and Goldman 2008). Initial attempts at AGR included larger sequence data sets, but specific othologs were removed whose TRIF sequence had evolved under different mechanisms. The confidence of our ten-species alignment for AGR was assessed using Guidence2 (Penn et al. 2010). A phylogenetic tree was generated for these species based on the topology as determined by Morgan et al. (2013). The species, Gibbon, was inserted manually onto this topology based on its positioning by the “Tree of life” online resource (Letunic and Bork 2011). The most likely marginally reconstructed sequence from the primate-rodent ancestral node was selected (fig. 1*B*).

The following versions of TRIF were synthesized by Invitrogen GeneART (https://www.thermofisher.com/uk/en/home/life-science/cloning/gene-synthesis/geneart-gene-synthesis.html#): 1) mTRIF, 2) ancestral TRIF (aTRIF), 3) ancestral TRIF whereby the amino acid residues predicted to be under selection were mutated to those residues present in either the extant mouse (maTRIF), or 4) the extant human (haTRIF). The mutagenesis strategy is detailed in figure 1*C*. The hTRIF mammalian expression construct, based on the pCMV2-FLAG vector, is described previously (Fitzgerald et al. 2003a). The synthetic *TRIF* genes were cloned into pCMV2-FLAG utilizing the same strategy as was used to engineer the hTRIF expression construct. Briefly, each gene was amplified from the original GeneArt vector using the primers oEMH1013 (5′-CTCACTATAGGGCGAATTGG-3′) and oEMH1014 (5′-GAAGGCCCATGAGGCCAGTTAATT-3′), subsequently digested with BamHI and SalI and cloned into the pCMV2-FLAG vector generating the plasmids pEMH1001 (mTRIF insert), pEMH1002 (aTRIF insert), pEMH1003 (maTRIF insert), and pEMH1004 (haTRIF insert). Two independent clones were sequence verified for each construct and used for subsequent phenotypic analysis.

### In Silico Protein Modeling and Mutational Analysis

To predict the structure of human and mTRIF protein, we first identified template proteins with high sequence similarity to the TRIF protein sequence, and whose structures are available. Then we constructed a theoretical structural model of the TRIF proteins from its amino acid sequence through homology modeling. To generate reliable models, we used MODELLER (Fiser and Sali 2003) software and the ModBase database. The method relies on an input sequence alignment between the amino acid sequence to be modeled and a template protein whose structure has been solved. For both and mTRIF, the sequence identity of the mouse sequence is 11%, and the template pdb used was 4k6j. We calculated dynamic flexibility index (dfi) scores of each residue in these homology modeled structures using our in-house program. To probe the effect of mutating the sites under selection in both the mouse and human predicted structures, we uploaded the corresponding pdb files into dezyme.com website and ran the systematic PoPMuSiC analysis which predicts changes in protein stability by all possible mutations at a given site using statistical potentials and neural networks (Dehouck et al. 2009).

### Cell Culturing and Transfection

The HEK 293 immortalized HEK cell line, expressing TLR4 was a gift from Prof Paul Moynagh at National University of Ireland, Maynooth. These cells were maintained in Dulbecco’s modified eagle’s medium (DMEM) supplemented with 10% (v/v) fetal bovine serum (FBS), and 2% (v/v) penicillin–streptomycin. Cells were incubated at 37 °C at in a CO_2_ incubator, and split every 3 days for a maximum of 20 passages.

The murine embryonic 3T3 cells were a gift from Dr Tomas Moore’s lab at University College Cork, and maintained in DMEM supplemented with 10% (v/v) FBS, 2 mM l-glutamine, 100 U/ml penicillin–streptomycin. Cells were incubated at 37 °C at in a CO_2_ incubator and split when they reached 70–80% confluency, after approximately 3 days, for a maximum of 20 passages. Both cell lines were transfected with plasmid DNA using lipofectamine 2000 (ThermoFisher scientific, 1668027). Briefly, 4 E + 04 of either HEK 293-TLR4 cells, or murine 3T3 cells were seeded in a 96-well plate and allowed to grow overnight to reach a 75–85% confluency. Approximately 50 ng of purified plasmid DNA was incubated with lipofectamine at a ratio of 1:4 (DNA [µg]: lipofectamine [µl]) for 20 min at room temperature and added to the cells. Cells were incubated overnight at 37 °C at in a CO_2_ incubator and analyzed the following day.

### RANTES ELISA Assay

Transfected HEK 293-TLR4 and murine 3T3 cells were analyzed for the expression and secretion of the cytokine RANTES. At the indicated time post-transfection, the supernatants from both HEK 293 and 3T3 cells were collected and 50 µl (undiluted) was analyzed using the DuoSet ELISA human RANTES (R&D systems DY278) and murine RANTES (R&D systems DY478) respectively in a 96-well format. As a control for RANTES production, mock-transfected cells were stimulated with 100 ng/ml LPS (Enzo life science, ALX-581-007-L002) for 10 h prior to ELISA analysis.

### Quantitative RT-PCR

To verify the expression of each transgene, total RNA was extracted from transfected cells and quantitative RT-PCR was undertaken. For RNA extraction, approximately 1 E + 06 both HEK 293-TLR4 and murine 3T3 cells were seeded in 6-well plates, transfected using lipofectamine as before, and at the indicated timepoints cells were harvested. Total RNA was prepared using the Macherey-Nagel RNA isolation kit (11922402), and cDNA was prepared using Biotin Tetro-cDNA synthesis kit (BIO-65042). Transcript abundance was determined using Applied Biosystem Fast SYBR green reagent (4385616). Supplementary table S3, Supplementary Material online contains a list of the primer sequences employed in this study.

## Supplementary Material

Supplementary data are available at *Genome Biology and Evolution* online.

## Supplementary Material

evab268_Supplementary_Data
