# Supplementary material for: Adaptive Evolution in TRIF Leads to Discordance between Human and Mouse Innate Immune Signaling
Source: Genome Biol Evol. 2021 Dec 6;13(12):evab268. doi: 10.1093/gbe/evab268 (PMC8691055; doi:10.1093/gbe/evab268)
Supplement: evab268_Supplementary_Data [file evab268_supplementary_data.zip › Post_review_Supp_figuresS1.pdf]

Figure S1 (5 pages)

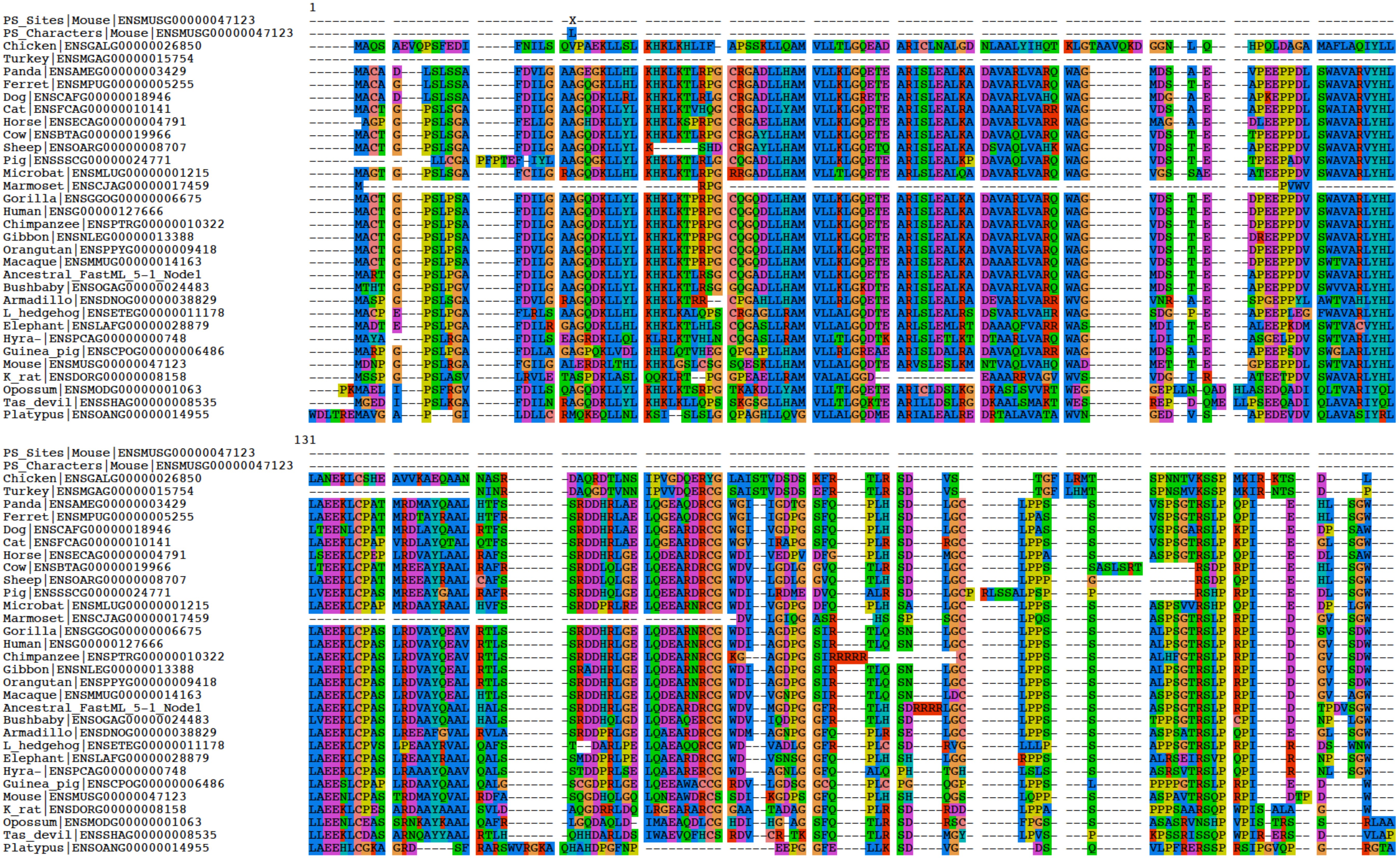

```
                                        261
PS_Sites|Mouse|ENSMUSG00000047123       ---------- ---------- ---------- ---------- ---------- ---------- ---------- ---------- ---------- ---------- ---------- ---------- ----------
PS_Characters|Mouse|ENSMUSG00000047123  ---------- ---------- ---------- ---------- ---------- ---------- ---------- ---------- ---------- ---------- ---------- ---------- ----------
Chicken|ENSGALG00000026850              -SGTQTLQSS GISDSF-TSL LISQSPTAIF CTPTPSCQS- ---SRLCEVS TS--DAGQPD GERQSHSLQE TGRASS-PS- --SHSRQDTN P-QVPHLGKT LQVSS---SR --L--SLP-- -IV----ETQ
Turkey|ENSMGAG00000015754               -SGPQTLQSS GISNSF-TNL LISQSPTAIF CMPTPSCES- ---SQLCEES TS--DTGQPD GERQSHSLQE TG---S-PR- --SHSTQDTN P-QVPHLGKT LQISS---SS --F---LP-- -IV----ETQ
Panda|ENSAMEG00000003429                -SRGRSLRST GSPASLASHL EISQSPTMPF LS---HHRSC HGPSKLCDEP QA--SLVPEP APPGCQEPQE VS---W-PP- --SIETV-SP PSVET----- ----A---NP PQAPR-LT-- -EL----VPD
Ferret|ENSMPUG00000005255               -SRGRSLRST GSPASLASNL EISQSPTLLF LS---HPRSC RGPSKLCDEP RA--SRVPEP APTGCQEPEE VS---W-PP- --LEETV-SP P-VLP----- ----S---SP --VPR-LT-- -EL----VPD
Dog|ENSCAFG00000018946                  -SRGRSLRST GSPASLASNL EISESPTMPF LS---RHRSC HEPSKLCDEP QA--SLVPEP APTGCQEPEE VS---W-PP- --SGETA-SP G-MLP----- ----N---SP --VP---TPP PDV----VPD
Cat|ENSFCAG00000010141                  -SRGCSLRST SSPASLASNL EISQSPTVPL LS---QHRGC RAPSKLCEEP RAGPGPEPEP APTGCQEPEE VS---W-PPD PSSGETA-GP Q-MAP----- ----D---SP --GPR-LP-- -EV----VPD
Horse|ENSECAG00000004791                -SRGCPLRSG GSRVSLASAL EISQSPTMRI LS---LQHGP HGP-RLCDEP RA--SVAPEP APTGCQEPEE MS---W-PP- --SGEPA-SP P-VLP----- ----S---HP --ALR-LP-- -ED----TPD
Cow|ENSBTAG00000019966                  -STACSLRST GSPASLGSNL EISQSPTMAL LS---VHHSH HGPSKLCDHP QA--SVVPEP APMGCQEPEE MS---W-PP- --SVEAA-DS P-VRP----- ----S---SP --GPG-LP-- -EV----TTD
Sheep|ENSOARG00000008707                -SRACSLRST GSPASLGSNL EISQSPTMAL LS---VHHSP HGPSKLCDHP QA--RVVPEP APLGCQEPEE MS---W-PP- --SVESA--- ---------- ---------- --GPG-LP-- -GG----PPD
Pig|ENSSSCG00000024771                  -SRGHSLRST GSPASLASNL EISQSPTMAL LS---LHHSP HGPSKLCDEP RA--SPVPEP APMGCQEPEE MS---W-PP- --SVEGA-NS P-VQS----- ----N---SP --VPR-DL-- -EV----AAD
Microbat|ENSMLUG00000001215             -SRGCSLRST GSPASLASSL EISQSPTMAL LG---QHHSP RGPSRLCDEP -----PEPEP VPAGFQEPEE MS---W-PT- --SEEIA-NT L-VPS----- ----D---SP --APG-LP-- -EE----ALD
Marmoset|ENSCJAG00000017459             -SQGRSLRST GSPASLTSNL EISQSPTMPL LS---LHRSP QGPSKLCDDP QA--SKVPEP VPGGCQEPEE MS---W-PP- --SGEIA-SP P-ELP----- ----S---SP --PPG-FC-- -KV----APD
Gorilla|ENSGGOG00000006675              -SQGCSLRST GSPASLASNL EISQSPTMPF LS---LHRSP HGPSKLCDDP QA--SLVPEP VPGGCQEPEE MS---W-PP- --SGEIA-SP P-ELP----- ----S---SP --PPG-LP-- -EV----APD
Human|ENSG00000127666                   -SQGCSLRST GSPASLASNL EISQSPTMPF LS---LHRSP HGPSKLCDDP QA--SLVPEP VPGGCQEPEE MS---W-PP- --SGEIA-SP P-ELP----- ----S---SP --PPG-LP-- -EV----APD
Chimpanzee|ENSPTRG00000010322           -SQGCSLRST GSPASLASNL EISQSPTVPF LS---LHRSP HGPSKLCDDP QA--SLVPEP VPGGCQEPEE MS---W-PP- --SGEIA-SP P-ELP----- ----S---SP --PPG-LP-- -EV----APD
Gibbon|ENSNLEG00000013388               -SQGCSLRST GSPASLASHL EISQSPTMPF LS---LHRSP HGPSRLCDDP QA--SLVPEP VPGGCQEPEE MS---W-PP- --SGEIA-SA P-ELP----- ----S---SP --PPG-LP-- -EV----APD
Orangutan|ENSPPYG00000009418            -SQGCSLRST GSPASLASNL EISQSPTMPF LS---LHRSP HRPSKLCDDP -A--S-VPEP VPGGCQEP-E MS---W-PP- --SGEIA-S- P-EL------ ---------- ---------- ----------
Macaque|ENSMMUG00000014163              -SQGRSLRST DSPASLASNL EISQSPTMPF LS---PHRSP HRPSKLCDDP QA--NLVLEP VPGGCQEPEE MS---W-PP- --SGETA-GP Q-ELP----- ----S---SP --PPA-LP-- -KV----APD
Ancestral_FastML_5-1_Node1              -SQGRSLRST GSPASLASNL EISQSPTMPF LS---PHRSP HGPSKLCDDP QA--STVPEP VPGGCQEPEE MS---W-PP- --SGEIA-GS P-ELP----- ----SHEISP --PPG-LP-- -EV----APD
Bushbaby|ENSOGAG00000024483             -SQGRSLRST DSPASLVSNL EISQSPTMPL LS---PHRSP HGPSKLCDSP QA--RTVPRP VPSSCQEPEE MT---W-PT- --SGEIA-GS P-ELP----- ---------- ---------- -EV----ALD
Armadillo|ENSDNOG00000038829            -SQGCPLRST DTPSSLASDL EISQSPTMPF IS---QHRAP PGPSKLCDEP LV--SPV--- -------PEE MS---W-PG- --SLQTA-G- ---------- --------AP --AAG-LP-- -TV----APD
L_hedgehog|ENSETEG00000011178           -SLAGPLRST GSPASLASNL EISQSPTMAL LT---QPTST PGPSKLSHQP AA--SLGGES VPMGRQEPEQ VS---W-PP- --SVQTD-PP Q-ELP----- ----D---SP --APQ-VP-- -VA----DPG
Elephant|ENSLAFG00000028879             -SQACSLKST DSPASLASNL EISQSPTMLF LT---QPHSP PRPSKLCDEP RD--SLRPEP TPMGCQEPEQ VN---W-PP- --SMETD-PP Q-EPP----- ----H---SP --APQ-LP-- -VV----APD
Hyra-|ENSPCAG00000000748                -SQAHSLKSA GSPASLASNL EISQSPTMQF NT---PPRSP PRPSKLCDQP QA--SPRPEL APMGCQEPEQ VS---W-PP- --STETD-PP Q-EQP----- ----Q---SP --PP------ -------APD
Guinea_pig|ENSCPOG00000006486           -SRGCSLRST GSLASLASAL EISQSPTAAF LS---HVRAP QGPSKLCEAP PG--S--TEP IPEGGQESQE MS---W-PT- --SAEIT-EP P-ELP----- ----S---I- ---------- ----------
Mouse|ENSMUSG00000047123                -SWGHTLHST NSTASLASHL EISQSPTLAF LS---SHHGT HGPSKLCNTP LD--TQEPQL VPEGCQEPEE IS---W-PP- --SVETS-VS L-GLP----- ----H---EI --S---VP-- -EV----SPE
K_rat|ENSDORG00000008158                -SRACSLRSS GSPGSVGAEL EISQSPTLPF LS---PHRAT HGPSKLPKAP VA-------- -PRGRQEPEE VS---W-PS- --SAEPL-DP D-RLH----- ----P---GP --G---HP-- -EV----ALA
Opossum|ENSMODG00000001063              ESSPQSLHST GSPASFASVL EISQSPTLPF LTYQPQKRSH RGPSKLCGDP HS--NSAQDP APQTSQELEE IN---L-SS- --SHPVA-SL H-EQP----- ----D---SL --A----S-- -VVSDPQPPN
Tas_devil|ENSSHAG00000008535            ESLPLTLRST GSPASFPSKL DISQSPTLAF FTCQPQKGGP KGPSKLCGDP QS--DSTEAS GP------EE TS---LWAC- --SDPAA-SL S-EQL----- ----D---SS --V----P-- -GVSGSQAPR
Platypus|ENSOANG00000014955             DASLRTLRSL GTPASFIGSL EISQSPTVPY FSTPPRQAPP RQPSKLCDGP VA--GSNQVP EGGARREPEG TN---R-PP- --EGPPP-AK P-SLP----- ----D---PR --T---LP-- ----------

                                        391
PS_Sites|Mouse|ENSMUSG00000047123       ---------- ---------- ---------- ---------- ---------- ---------- ---------- -----X---- ---------- ---------- ---------X ---------- ----------
PS_Characters|Mouse|ENSMUSG00000047123  ---------- ---------- ---------- ---------- ---------- ---------- ---------- -----S---- ---------- ---------- ---------D ---------- ----------
Chicken|ENSGALG00000026850              LPILGAVGQP VESNDISSTV IAEPQVPKES RDQKQKLSTS ---------- ---------- ---------- ---------- ---------- LPYSRMTINT GPACIPIEDS Y------IPA -GTSNSAPAS
Turkey|ENSMGAG00000015754               LPTLGAVGQP VESNDISSTV IAEPQVPKKS RDQKQKLSTS ---------- ---------- ---------- ---------- ---------- LPYSRTTINT GPACIPIEDS Y------IPA -GTSNSAAAS
Panda|ENSAMEG00000003429                ASPAGQPDPP RA-------- ---LEISTHY PVECTDALAA PESL------ -S---L-PS- ---------- -------RNT WP-DKGQSP- FPLP------ ------EEDT ASQVASAS-- --PP-PPAP-
Ferret|ENSMPUG00000005255               ASHAGQPDPP RA-------- ---LEISTHY PVECTEELGG PKSL------ -S---L-AS- ---------- -------RNP CH-DKDQTP- LPLP------ ------VEDT ASQVASPR-- --PPAPPAP-
Dog|ENSCAFG00000018946                  ASLSDQLDPP KA-------- ---GQMGTHY PVECTEMLAA PSSL------ -S---L-PS- ---------- -------GNA RP-VKDQTP- LPLP------ ------VEDT ASQLPNPS-- --PP-PPSA-
Cat|ENSFCAG00000010141                  ASPTGQPDAP KA-------- ---LESGTRY PVECSHALAA PASL------ -P---L-PP- ---------- -------KTT CP-DKDQTP- LSLP------ ------VEDT ASQRTPPC-- --PP-TPSA-
Horse|ENSECAG00000004791                V-PAGLADPP KA-------- ---PETGTHY PVECTEVSAA PPSL------ -A---S-PS- ---------- -------R-- -P-VRDPTP- PQLP------ ------AEDA ARL--PPK-- PCPP-TPSA-
Cow|ENSBTAG00000019966                  ACPASPHDPP EV-------- ---PEISAHY PVECTDVPAA PKSL------ -P---S-PS- ---------- -------KNT CL-VTDQTP- VQLS------ ------EEDT AYLSAQPR-- --PP-TPSV-
Sheep|ENSOARG00000008707                ACPASPHNPP EV-------- ---PEISAHY PVECTDVPAA PKSL------ -P---S-PS- ---------- -------KNA CL-VTDQTP- VQHS------ ------EEDT AYLSAQPR-- --PP-TPSV-
Pig|ENSSSCG00000024771                  ASPASLPEAP EA-------- ---PETSTHY PVECTEEPAA PTTL------ -P---S-PS- ---------- -------RNA SPDVTDQKPP LHLS------ ------EEDT TYPTAQPH-- --PP-TPSV-
Microbat|ENSMLUG00000001215             ASPAGPSNAA EA-------- ---PGTSTHY PVECTEVTPA PTSL------ -P---S-PC- ---------- -------RNT GP-AEDQVP- LQFP------ ------VEDT PSPPAQPC-- --PP-TSSA-
Marmoset|ENSCJAG00000017459             ATSTGLLDTP EA-------- ---PETSTQF PVECTEGSAG PQSL------ -P---L-PI- L-----E--- -----PPQNP CP-VKGQTS- PQLS------ ------VEDT TSPNTKP-S- ---P-SPTT-
Gorilla|ENSGGOG00000006675              ATSTGLPDTP AA-------- ---PETSTNY PVECTEGSAG PQSL------ -P---L-PI- L-----E--- -----PVKNP CS-VKDQTP- LQLS------ ------VEDT TSPNTKPC-- --PP-TPTT-
Human|ENSG00000127666                   ATSTGLPDTP AA-------- ---PETSTNY PVECTEGSAG PQSL------ -P---L-PI- L-----E--- -----PVKNP CS-VKDQTP- LQLS------ ------VEDT TSPNTKPC-- --PP-TPTT-
Chimpanzee|ENSPTRG00000010322           ATSTGLPDTP AA-------- ---PETSTNY PVECTEGSAG PQSL------ -P---L-PI- L-----E--- -----PVKNP CS-VKDQTP- LQLS------ ------VEDT TSPNTKPC-- --PP-TPTT-
Gibbon|ENSNLEG00000013388               ATSTGLPDTP TA-------- ---PETSTNY PVECTEGSAG PQSL------ -P---L-PM- L-----E--- -----PVKNS CS-VKDQT-- ---------- ---------- ---------- -----TPTT-
Orangutan|ENSPPYG00000009418            ---------- ---------- ---------- ---------- ---------- ---------- ---------- ---------- ---------- --LS------ ------VEDT TSP-TKPC-- --PP-TPTK-
Macaque|ENSMMUG00000014163              TTSTGLPETP TA-------- ---PETSTHY PVECTEESAG GQSL------ -P---S-PI- V-----E--- -----PVKNP CP-VKHQSP- LQLS------ ------VEDT TSPDTKPC-- --PP-TPTT-
Ancestral_FastML_5-1_Node1              AASTGLPDTP AA-------- ---PETSTHY PVECTEVSAA SRSL------ -S---L-PI- L-----E--- -----PVENP RP-VKDQTP- PQLS------ ------VEDD SLQTTSPDTK PCPP-TPST-
Bushbaby|ENSOGAG00000024483             PVPAGLPDTP QT-------- ---PGTSTHY PVQCTEVSAA SGSL------ -S---S-PI- L-----E--- -----PVGHP RP-DKYQAP- HQLS------ ------KEDT TTPDTKSC-- --PP-TPSI-
Armadillo|ENSDNOG00000038829            PSPEGLLGPS AV-------- ---PETSSHR PVDCTEVSEA PNSL------ -P---S-PA- P-----E--- -----PPENP RP-ANARTP- LPLS------ ------VEDT TSQEAMLC-- --PP-TPSA-
L_hedgehog|ENSETEG00000011178           P---GLPHPS AP-------- ---PEPRAHC PVECSEVSEG PQSLS----- -PPTP----- ------E--- -----PPGD- -P-SQDRTL- FSGP------ ------GGDT ASQKTRLKS- ---P-PPPA-
Elephant|ENSLAFG00000028879             PSPAGLPNPS TP-------- ---LASRAHY PEECTEVSVA PQSL------ -PSTPQSLPS PVL---D--- -----PPDN- -P-TKNGTS- VPLS------ ------VEDT ASQKSKQS-- --PP-PPSA-
Hyra-|ENSPCAG00000000748                PSPPDLLDPA PP-------- ---LESGAHS PEESE--SVV SQPL------ -LPPPQS-SS P--------- ---------- ---------- ---------- ---------T AA-------- --PP-PLRQ-
Guinea_pig|ENSCPOG00000006486           LTPSGDPDCP AA-------- ---------- PVECTDTSTA PKAL------ -S---L-PN- L-----E--- -----PRESL HP-VQDQTA- PPAS------ ------AEDT TSPESNPS-- --PP-ASSA-
Mouse|ENSMUSG00000047123                EASPILPDAL AA-------- ---PDTSVHC PIECTELSTN SRSP------ -L---T-ST- T-----E--- -----SVGKQ WP-ITSQRS- PQVP------ ------VGDD SLQNTTSSS- --PP-AQP--
K_rat|ENSDORG00000008158                SVSS---NST VP-------- ---PDGGSQG SLERTGECRD PPSL------ -L---S-AS- L-----D--- -----PTENP CP-GEDHTT- LR-S------ ------PEDA TRTNAQPC-- --PP-TP-A-
Opossum|ENSMODG00000001063              PSISPPLITP VD-------- ---PETSLHS STECSEALSD TQSL-QPASG KP---S-EA- L--ISKESSG LLPGLPPMSV PP-VGEGEP- TQFS------ ------VQES N-----CSE- -EVSD-----
Tas_devil|ENSSHAG00000008535            PSTSPSPAPS VA-------- ---PETSRH- SVECNETLSD PTPP-ECVST KP---A-EI- L-----ESFG VLPELPPVSV LL-AREEKA- TLLS------ ------VQEG D-----LPK- -KTSS-----
Platypus|ENSOANG00000014955             ---PGAASGP RC-------- ---PAAERRR PTPGTGAAGD ---------- ---------- ---------- ---------- ---------- LELS------ ------GED- ---------- ----------
```

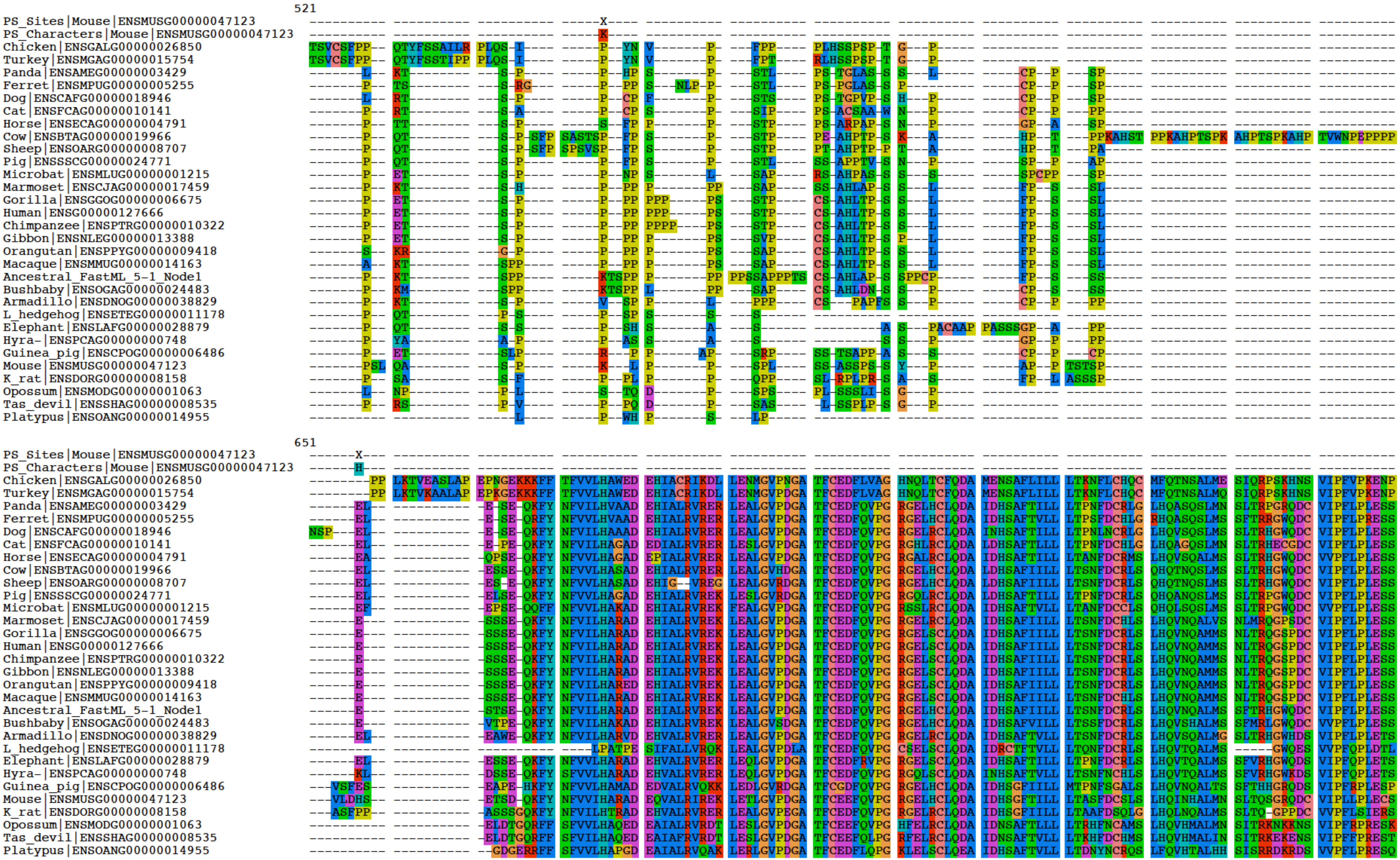

521
PS_Sites|Mouse|ENSMUSG00000047123
PS_Characters|Mouse|ENSMUSG00000047123
Chicken|ENSGALG00000026850
Turkey|ENSMGAG00000015754
Panda|ENSAMEG00000003429
Ferret|ENSMPUG00000005255
Dog|ENSCAFG00000018946
Cat|ENSFCAG00000010141
Horse|ENSECAG00000004791
Cow|ENSBTAG00000019966
Sheep|ENSOARG00000008707
Pig|ENSSSCG00000024771
Microbat|ENSMLUG00000001215
Marmoset|ENSCJAG00000017459
Gorilla|ENSGGOG00000006675
Human|ENSG00000127666
Chimpanzee|ENSPTRG00000010322
Gibbon|ENSNLEG00000013388
Orangutan|ENSPPYG00000009418
Macaque|ENSMMUG00000014163
Ancestral_FastML_5-1_Node1
Bushbaby|ENSOGAG00000024483
Armadillo|ENSDNOG00000038829
L_hedgehog|ENSETEG00000011178
Elephant|ENSLAFG00000028879
Hyra-|ENSPCAG00000000748
Guinea_pig|ENSCPOG00000006486
Mouse|ENSMUSG00000047123
K_rat|ENSDORG00000008158
Opossum|ENSMODG00000001063
Tas_devil|ENSSHAG00000008535
Platypus|ENSOANG00000014955
651

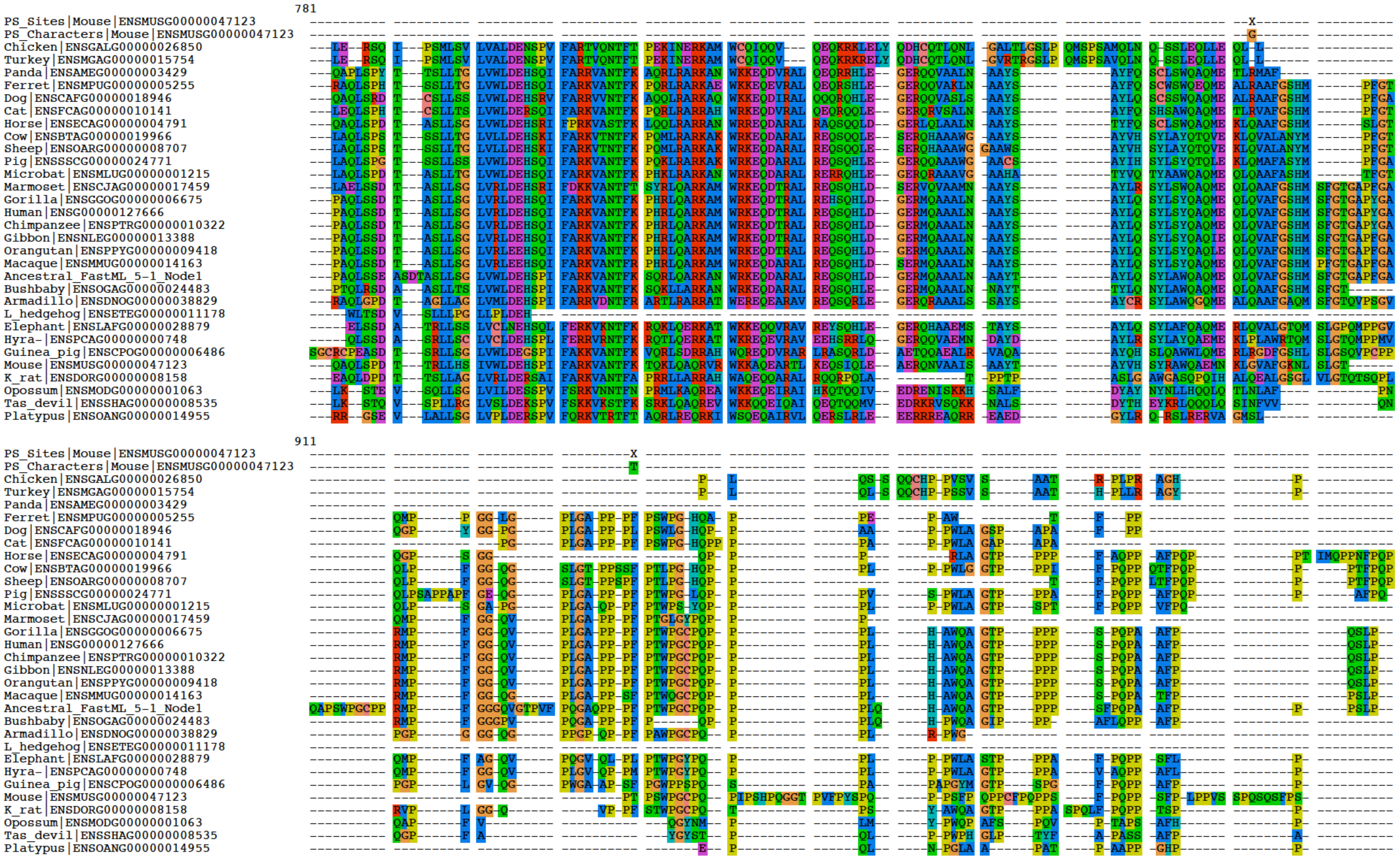

781
PS_Sites|Mouse|ENSMUSG00000047123
PS_Characters|Mouse|ENSMUSG00000047123
Chicken|ENSGALG00000026850
Turkey|ENSMGAG00000015754
Panda|ENSAMEG00000003429
Ferret|ENSMPUG00000005255
Dog|ENSCAFG00000018946
Cat|ENSFCAG00000010141
Horse|ENSECAG00000004791
Cow|ENSBTAG00000019966
Sheep|ENSOARG00000008707
Pig|ENSSSCG00000024771
Microbat|ENSMLUG00000001215
Marmoset|ENSCJAG00000017459
Gorilla|ENSGGOG00000006675
Human|ENSG00000127666
Chimpanzee|ENSPTRG00000010322
Gibbon|ENSNLEG00000013388
Orangutan|ENSPPYG00000009418
Macaque|ENSMMUG00000014163
Ancestral_FastML_5-1_Node1
Bushbaby|ENSOGAG00000024483
Armadillo|ENSDNOG00000038829
L_hedgehog|ENSETEG00000011178
Elephant|ENSLAFG00000028879
Hyra-|ENSPCAG00000000748
Guinea_pig|ENSCPOG00000006486
Mouse|ENSMUSG00000047123
K_rat|ENSDORG00000008158
Opossum|ENSMODG00000001063
Tas_devil|ENSSHAG00000008535
Platypus|ENSOANG00000014955
911

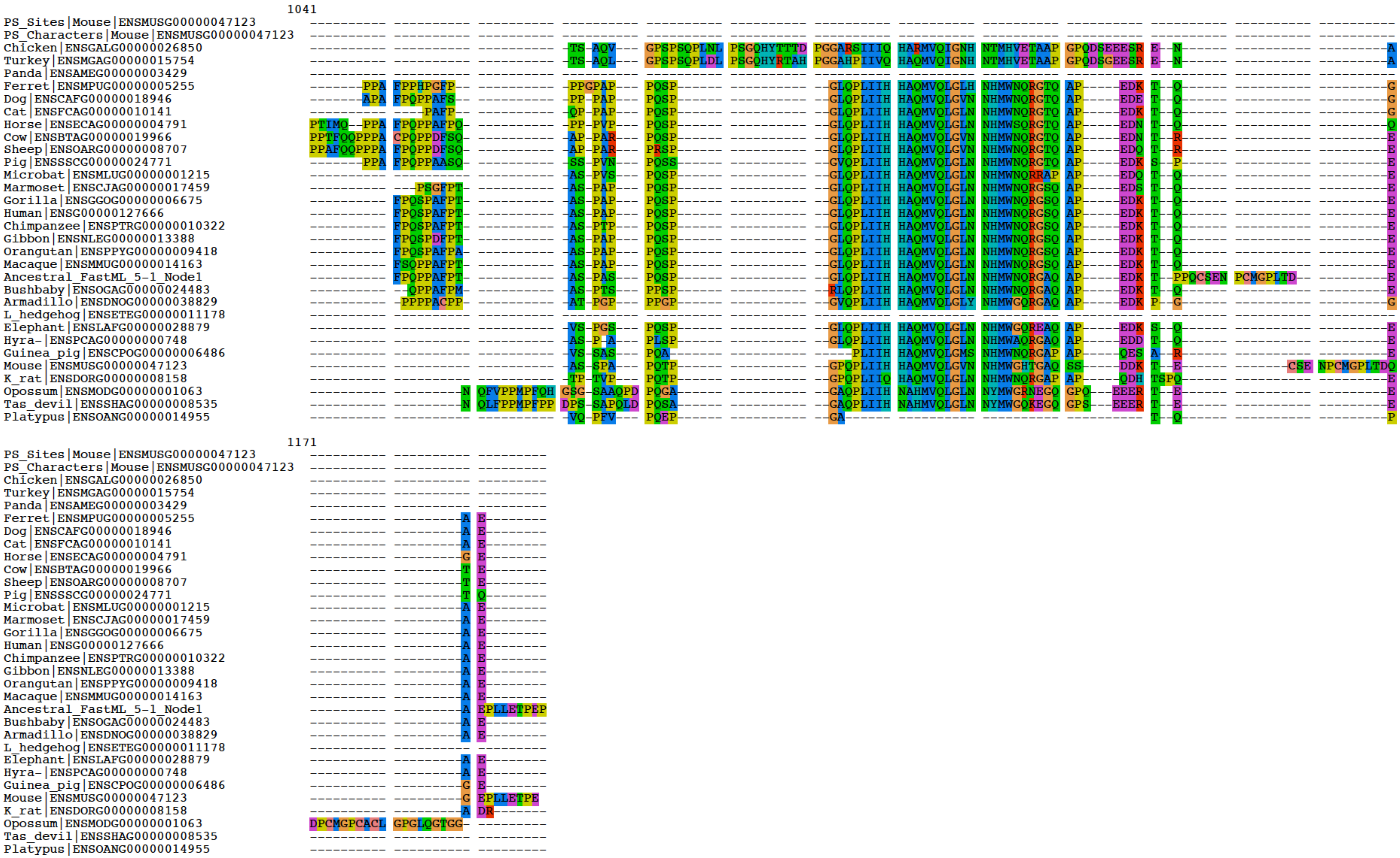

Figure S1 The PRANK amino acid alignment of all the TRIF homologues from the 27 different mammals used in the positive selection analysis with CodeML. Also shown is the position of the positively selected sties (PS_Sites) detected in the mouse lineage and their corresponding amino acid (PS-Characters). This alignment had an overall confidence score of 0.883 using Guidance2 software..
