# Supplementary material for: Adaptive Evolution in TRIF Leads to Discordance between Human and Mouse Innate Immune Signaling
Source: Genome Biol Evol. 2021 Dec 6;13(12):evab268. doi: 10.1093/gbe/evab268 (PMC8691055; doi:10.1093/gbe/evab268)
Supplement: evab268_Supplementary_Data [file evab268_supplementary_data.zip › Post_review_Supp_figuresS2.pdf]

Figure S2 (2 pages)

```
                 1
Marmoset         M--------------------------------RPG-------------- --------------------------------------PVWV--------
Guinea_pig       MARPGPSLPGAFDLLAGAGPQKLVDLRHRLQTVHEGQPGAPLLHAMVLLR LGREAEARISLDALRADAVAQLVARRWAGMDSAEAPEEPSDVSWGLARLY
Gorilla          MACTGPSLPSAFDILGAAGQDKLLYLKHKLKTPRPGCQGQDLLHAMVLLK LGQETEARISLEALKADAVARLVARQWAGVDSTEDPEEPPDVSWAVARLY
Macaque          MACTGPSLPSAFDILGAAGQDKLLYLKHKLKTPRPGCQGQDLLHAMVLLK LGQETEARISLEALKADAAARLVARQWAGVDSTEDPEEPPDVSWTVARLY
Mouse            MDNPGPSLRGAFGILGALERDRLTHLKHKLGSLCSGSQESKLLHAMVLLA LGQDTEARVSLESLKMNTVAQLVAHQWADMETTEGPEEPPDLSWTVARLY
Gibbon           MACTGPSLPSAFDILGAAGQDKLLYLKHKLKTPRPGCQGQDLLHAMVLLK LGQETEARISLEALKADAVARLVARQWAGVDSTEDREEPPDVSWAVARLY
Bushbaby         MTHTGPSLPGVFDILGAAGQDKLLYLKHKLKTLRSGGQGADLLHAMVLLK LGKDTEARISLEALKADAVARLVARQWAGMDSTEAPEEPPDVSWVVARLY
Chimpanzee       MACTGPSLPSAFDILGAAGQDKLLYLKHKLKTPRPGCQGQDLLHAMVLLK LGQETEARISLEALKADAVARLVARQWAGVDSTEDPEEPPDVSWAVARLY
Orangutan        MACTGPSLPSAFDVLGAAGQDKLLYLKHKLKTPRPGCQGQDLLHAMVLLK LGQETEARISLEALKADAVARLVARQWAGVDSTEDPEEPPDVSWAVARLY
Human            MACTGPSLPSAFDILGAAGQDKLLYLKHKLKTPRPGCQGQDLLHAMVLLK LGQETEARISLEALKADAVARLVARQWAGVDSTEDPEEPPDVSWAVARLY
Ancestral_Node_N1 MARTGPSLPGAFDILGAAGQDKLLYLKHKLKTLRSGCQGADLLHAMVLLK LGQETEARISLEALKADAVARLVARQWAGMDSTEAPEEPPDVSWAVARLY

                 101
Marmoset         ----------------------------------------------DVLG IQGASR-HSSP----SGCLPQSSASPSGTRSLPRPID--GVSGWSQGRSL
Guinea_pig       HLLAEESLCPAPLRDAAYQAALQALGSCGDPRLGELQEEAWACCGRDVLG DSGGCQPLCPG----QGPLPPSLPPPPGTRSLPRPIE--D---WSRGCSL
Gorilla          HLLAEEKLCPASLRDVAYQEAVRTLSSRDDHRLGELQDEARNRCGWDIAG DPGSIRTLQSN----LGCLPPSSALPSGTRSLPRPID--SVSDWSQGCSL
Macaque          HLLAEEKLCPASLRDVAYQEALHTLSSRDDHRLGELQDEARNRCGWDVVG NPGSIRTLQSN----LDCLPPSSASPSGTRSLPRPID--GVAGWSQGRSL
Mouse            HLLAEENLCPASTRDMAYQVALRDFASQGDHQLGQLQNEAWDRCSSDIKG DPSGFQPLHSH----QGSLQPPSASPAVTRSQPRPIDTPD---WSWGHTL
Gibbon           HLLAEERLCPASLRDVAYQEALRTLSSRADHRLGELQDEARNRCGWDIAG DPGSIRTLQSN----LGCLPPSSALPSGTRSLPRPID--GVSDWSQGCSL
Bushbaby         HLLVEEKLCPASLRDAAYQAALHALSSRDDHQLGDLQDEAQERCGWDVIQ DPGGFRTLHSD----LGCLPPSSTPPSGTRSLPCPID--NPLGWSQGRSL
Chimpanzee       HLLAEEKLCPASLRDVAYQEAVRTLSSRDDHRLGELQDEARNRCGKG-AG DPGSIR-----RRRR--CLPPSSALHFGTRSLPRPID--GVSDWSQGCSL
Orangutan        HLLAEEKLCPASLRDVAYQEALRTLSSRDDHRLGELQDEARNRCGWDIAG DPGSIRTLQSN----LGCLPPSSALPSGTRSLPRPID--GVSDWSQGCSL
Human            HLLAEEKLCPASLRDVAYQEAVRTLSSRDDHRLGELQDEARNRCGWDIAG DPGSIRTLQSN----LGCLPPSSALPSGTRSLPRPID--GVSDWSQGCSL
Ancestral_Node_N1 HLLAEEKLCPASLRDVAYQAALHALSSRDDHRLGELQDEARDRCGWDVMG DPGGFRTLHSDRRRRLGCLPPSSASPSGTRSLPRPIDTPDVSGWSQGRSL

                 201
Marmoset         RSTGSPASLTSNLEISQSPTMPLLSLHRSPQGPSKLCDDPQASKVPEPVP GGCQEPEEMSWPPSGEIASPPELPS---SPPPGFCKVAPDATSTGLLDTP
Guinea_pig       RSTGSLASLASALEISQSPTAAFLSHVRAPQGPSKLCEAPPGSTEP--IP EGGQESQEMSWPTSAEITEPPELP----S----ILTPSGD------PDCP
Gorilla          RSTGSPASLASNLEISQSPTMPFLSLHRSPHGPSKLCDDPQASLVPEPVP GGCQEPEEMSWPPSGEIASPPELPS---SPPPGLPEVAPDATSTGLPDTP
Macaque          RSTDSPASLASNLEISQSPTMPFLSPHRSPHRPSKLCDDPQANLVLEPVP GGCQEPEEMSWPPSGETAGPQELPS---SPPPALPKVAPDTTSTGLPETP
Mouse            HSTNSTASLASHLEISQSPTLAFLSSHHGTHGPSKLCNTPLDTQEPQLVP EGCQEPEEISWPPSVETSVSLGLP-HEIS----VPEVSPEEASPILPDAL
Gibbon           RSTGSPASLASHLEISQSPTMPFLSLHRSPHGPSRLCDDPQASLVPEPVP GGCQEPEEMSWPPSGEIASAPELPS---SPPPGLPEVAPDATSTGLPDTP
Bushbaby         RSTDSPASLVSNLEISQSPTMPLLSPHRSPHGPSKLCDSPQARTVPRPVP SSCQEPEEMTWPTSGEIAGSPELP-----------EVALDPVPAGLPDTP
Chimpanzee       RSTGSPASLASNLEISQSPTVPFLSLHRSPHGPSKLCDDPQASLVPEPVP GGCQEPEEMSWPPSGEIASPPELPS---SPPPGLPEVAPDATSTGLPDTP
Orangutan        RSTGSPASLASNLEISQSPTMPFLSLHRSPHRPSKLCDDP-AS-VPEPVP GGCQEPE-MSWPPSGEIAS-PEL---------------------------
Human            RSTGSPASLASNLEISQSPTMPFLSLHRSPHGPSKLCDDPQASLVPEPVP GGCQEPEEMSWPPSGEIASPPELPS---SPPPGLPEVAPDATSTGLPDTP
Ancestral_Node_N1 RSTGSPASLASNLEISQSPTMPFLSPHRSPHGPSKLCDDPQASTVPEPVP GGCQEPEEMSWPPSGEIAGSPELPSHEISPPPGLPEVAPDAASTGLPDTP

                 301
Marmoset         EAPETSTQFPVECTEGSAGPQSLPLPILEPPQNPCPVKGQTSPQLSVED- ---TTSPNTKP-SPSPTTPKTSHP---P--PPPP-SAP----SSAHLAPS
Guinea_pig       AA-------PVECTDTSTAPKALSLPNLEPRESLHPVQDQTAPPASAED- ---TTSPESNPSPPASSAPETSLP---RPP--APSR---PSSTSAPPASS
Gorilla          AAPETSTNYPVECTEGSAGPQSLPLPILEPVKNPCSVKDQTPLQLSVED- ---TTSPNTKPCPPTPTTPETSPP---PP-PPPPSSTP----CSAHLTPS
Macaque          TAPETSTHYPVECTEESAGGQSLPSPIVEPVKNPCPVKHQSPLQLSVED- ---TTSPDTKPCPPTPTTAKTSPP---P--PPPPSSAP----CSAHLTPS
Mouse            AAPDTSVHCPIECTELSTNSRSPLTSTTESVGKQWPITSQRSPQVPVGDD SLQNTTSSSPPAQPPSLQASPKLP---PSPLSSASS---PSSYPAPPTST
Gibbon           TAPETSTNYPVECTEGSAGPQSLPLPMLEPVKNSCSVKDQT--------- --------------TPTTPETSPP---P---PPPSSVP----CSAHLTPS
Bushbaby         QTPGTSTHYPVQCTEVSAASGSLSSPILEPVGHPRPDKYQAPHQLSKED- ---TTTPDTKSCPPTPSIPKMSPPKTSP--PLPP-SAP----CSAHLDNS
Chimpanzee       AAPETSTNYPVECTEGSAGPQSLPLPILEPVKNPCSVKDQTPLQLSVED- ---TTSPNTKPCPPTPTTPETSPP---PPPPPPPSSTP----CSAHLTPS
Orangutan        --------------------------------------------LSVED- ---TTSP-TKPCPPTPTKSKRGPP---P---PPPSSAP----CSAHLTPS
Human            AAPETSTNYPVECTEGSAGPQSLPLPILEPVKNPCSVKDQTPLQLSVED- ---TTSPNTKPCPPTPTTPETSPP---PP-PPPPSSTP----CSAHLTPS
Ancestral_Node_N1 AAPETSTHYPVECTEVSAASRSLSLPILEPVENPRPVKDQTPPQLSVEDD SLQTTSPDTKPCPPTPSTPKTSPPKTSPPPPPPPSSAPPPTSCSAHLAPS
```

```
                   401
Marmoset           SL---FPSSLESSSEQKFYNFVILHARADEHIALRVREKLEALGVPDGAT FCEDFQVPGRGELRCLQDAIDHSAFIILLLTSNFDCHLSLHQVNQALVSN
Guinea_pig         CPPCPVSFESE-APEHKFYNFVVLHAMADEDVALRVQKKLEDLGVRDGAT FCGDFQVPGRGELHCLQDAIDHSGFIILLMTPNFSGALSLHQVNQALTSS
Gorilla            SL---FPSSLESSSEQKFYNFVILHARADEHIALRVREKLEALGVPDGAT FCEDFQVPGRGELSCLQDAIDHSAFIILLLTSNFDCRLSLHQVNQAMMSN
Macaque            SL---FPSSLESSSEQKFYNFVILHARADEHIALRVREKLEALGVPDGAT FCEDFQVPGRGELSCLQDAIDHSAFIILLLTSNFDCHLSLHQVNQAMMSN
Mouse              SP---VLDHSE-TSDQKFYNFVVIHARADEQVALRIREKLETLGVPDGAT FCEEFQVPGRGELHCLQDAIDHSGFTILLLTASFDCSLSLHQINHALMNS
Gibbon             PL---FPSSLESSSEQKFYNFVILHARADEHIALRVREKLEALGVPDGAT FCEDFQVPGRGELSCLQDAIDHSAFIILLLTSNFDCRLSLHQVNQAMMSN
Bushbaby           SP---CPSSSEVTPEQKFYNFVILHAKADEHIALRVREKLEALGVSDGAT FCEDFQVPGRGELHCLQDAIDHSAFVILLLTSSFDCRLSLHQVSHALMSS
Chimpanzee         SL---FPSSLESSSEQKFYNFVILHARADEHIALRVREKLEALGVPDGAT FCEDFQVPGRGELSCLQDAIDHSAFIILLLTSNFDCRLSLHQVNQAMMSN
Orangutan          SL---FPSSLESSSEQKFYNFVILHAREDEHIALRVREKLEALGVPDGAT FCEDFQVPGRGELSCLQDAIDHSAFIILLLTSNFDCRLSLHQVNQAMMSN
Human              SL---FPSSLESSSEQKFYNFVILHARADEHIALRVREKLEALGVPDGAT FCEDFQVPGRGELSCLQDAIDHSAFIILLLTSNFDCRLSLHQVNQAMMSN
Ancestral_Node_N1  SPPCPFPSSSESTSEQKFYNFVILHARADEHIALRVREKLEALGVPDGAT FCEDFQVPGRGELHCLQDAIDHSAFIILLLTSNFDCRLSLHQVNQALMSS

                   501
Marmoset           LMRQGPSDCVIPFLPLESSLAELSS---DTASLLSGLVRLDEHSRIFDKK VANTFTSYRLQARKAMWRKEQDTRALREQSQHLDSERVQVAAMNAAYSAY
Guinea_pig         FTHHGRQDSVIPFRPLESPSGCRCPEASDTSRLLSGLVWLDEGSPIFAKK VANTFKVQRLSDRRAHWQREQDVRARLRASQRLDAETQQAEALRVAQAAY
Gorilla            LTRQGSPDCVIPFLPLESSPAQLSS---DTASLLSGLVRLDEHSQIFARK VANTFKPHRLQARKAMWRKEQDTRALREHSQHLDGERMQAAALNAAYSAY
Macaque            LTRQGSPDCVIPFLPLESSPAQLSS---DTASLLSGLVRLEEHSQIFARK VANTFRPHRLQARKAMWRKEQDARALREQSQHLDSERMQAAALNAAYSAY
Mouse              LTQSGRQDCVIPLLPLECSQAQLSP---DTTRLLHSIVWLDEHSPIFARK VANTFKTQKLQAQRVRWKKAQEARTLKEQSIQLEAERQNVAAISAAYTAY
Gibbon             LTRQGSPDCVIPFLPLESSPAQLSS---DTASLLSGLVRLDEHSQIFARK VANTFKPHRLQARKAMWRKEQDTRALREQSQHLDGERMQAAALNAAYSAY
Bushbaby           FMRLGWQDCVVPFLPLESSPTQLRS---DAASLLTSLVWLDEHSPIFARK VANTFKSQKLLARKANWRKEQDARALREQSQHLEGERMQAAALNNAYTTY
Chimpanzee         LTRQGSPDCVIPFLPLESSPAQLSS---DTASLLSGLVRLDEHSQIFARK VANTFKPHRLQARKAMWRKEQDTRALREQSQHLDGERMQAAALNAAYSAY
Orangutan          LTRQGSPDCVIPFLPLESSPAQLSS---DTASLLSGLVRLEEHSQIFARK VANTFKPHRLQARKAMWRKEQDTRALREQSQHLDGERMQAAALNAAYSAY
Human              LTRQGSPDCVIPFLPLESSPAQLSS---DTASLLSGLVRLDEHSQIFARK VANTFKPHRLQARKAMWRKEQDTRALREQSQHLDGERMQAAALNAAYSAY
Ancestral_Node_N1  FTRHGWQDCVIPFLPLESSPAQLSSEASDTASLLSGLVWLDEHSPIFARK VANTFKSQRLQARKANWRKEQDARALREQSQHLDGERMQAAALNAAYTAY

                   601
Marmoset           LRSYLSWQAQMEQLQAAFGSHMSFGTGAPFGA----------QMPFGG-Q V-----PLGA-PPPFPTGLGYPQPPP------------------------
Guinea_pig         QHSLQAWWLQMERLRGDFGSHLSLGS------QVP----CPPPGPLGV-Q G-----PWGA-APSFPGWPPSPQSP--------------A-PAPGYMGTP
Gorilla            LQSYLSYQAQMEQLQVAFGSHMSFGTGAPYGA----------RMPFGG-Q V-----PLGA-PPPFPTWPGCPQPPPL-HAWQAGTPPPPS-PQPAAFP-Q
Macaque            LQSYLSYQAQMEQLQVAFGSHMPFGTGAPFGA----------RMPFGG-Q G-----PLGA-PPSFPTWQGCPQPPPL-HAWQAGTPPPPS-PQPATFP-P
Mouse              VHSYRAWQAEMNKLGVAFGKNLSLGT------PTPSWPGCPQPIPSHP-Q GGTPVFPYSPQPPSFPQPPCFPQPP--------------SFPQPPSFPLP
Gibbon             LQSYLSYQAQIEQLQVAFGSHMSFGTGAPFGA----------RMPFGG-Q V-----PLGA-PPPFPTWPGCPQPPPL-HAWQAGTPPPPS-PQPAAFP-Q
Bushbaby           LQNYLAWQAQMEQLQAAFGSHMSFGT----------------RMPFGGGP V-----PQGA-PPPFP------QPPPLQHPWQAGIPPPAF-LQPPAFP--
Chimpanzee         LQSYLSYQAQMEQLQVAFGSHMSFGTGAPYGA----------RMPFGG-Q V-----PLGA-PPPFPTWPGCPQPPPL-HAWQAGTPPPPS-PQPAAFP-Q
Orangutan          LQSYLSYQAQLEQLQVAFGNHMSFGTGAPFGA----------RMPFGG-Q V-----PLGA-PPPFPTWPGCPQPPPL-HAWQAGTPPPPS-PQPAAFP-Q
Human              LQSYLSYQAQMEQLQVAFGSHMSFGTGAPYGA----------RMPFGG-Q V-----PLGA-PPPFPTWPGCPQPPPL-HAWQAGTPPPPS-PQPAAFP-Q
Ancestral_Node_N1  LQSYLAWQAQMEQLQVAFGSHMSFGTGAPFGAQAPSWPGCPPRMPFGGGQ VGTPVFPQGAQPPPFPTWPGCPQPPPLQHAWQAGTPPPPSFPQPAAFPPP

                   701
Marmoset           ------PSGFPTASPAPPQSPGLQPLIIHHAQMVQLGLNNHMWNQRGSQA PEDST--Q------------EAE--------
Guinea_pig         SPGFPQPPAFPPVSSASPQAP----LIIHHAQMVQLGMSNHMWNQRGAPA PQESA--R------------EGE--------
Gorilla            SLPFPQSPAFPTASPAPPQSPGLQPLIIHHAQMVQLGLNNHMWNQRGSQA PEDKT--Q------------EAE--------
Macaque            SLPFSQPPAFPTASPAPPQSPGLQPLIIHHAQMVQLGLNNHMWNQRGSQA PEDKT--Q------------EAE--------
Mouse              PVSSPQSQSFPSASSPAPQTPGPQPLIIHHAQMVQLGVNNHMWGHTGAQS SDDKT--ECSENPCMGPLTDQGEPLLETPE-
Gibbon             SLPFPQSPDFPTASPAPPQSPGLQPLIIHHAQMVQLGLNNHMWNQRGSQA PEDKT--Q------------EAE--------
Bushbaby           -----QPPAFPMASPTSPPSPRLQPLIIHHAQMVQLGLNNHMWNQRGAQA PEDKT--Q------------EAE--------
Chimpanzee         SLPFPQSPAFPTASPTPPQSPGLQPLIIHHAQMVQLGLNNHMWNQRGSQA PEDKT--Q------------EAE--------
Orangutan          SLPFPQSPAFPAASPAPPQSPGLQPLIIHHAQMVQLGLNNHMWNQRGSQA PEDKT--Q------------EAE--------
Human              SLPFPQSPAFPTASPAPPQSPGLQPLIIHHAQMVQLGLNNHMWNQRGSQA PEDKT--Q------------EAE--------
Ancestral_Node_N1  SLPFPQPPAFPTASPASPQSPGLQPLIIHHAQMVQLGLNNHMWNQRGAQA PEDKTPPQCSENPCMGPLTDEAEPLLETPEP
```

Figure S2 Amino acids sequence alignment of 10 species used in FastML prediction of the ancestral TRIF sequence (identified here as Ancestral_node_N1) Confidence score of 0.947 was obtained for this alignment using Guidance2 software.
