# Supplementary material for: Adaptive Evolution in TRIF Leads to Discordance between Human and Mouse Innate Immune Signaling
Source: Genome Biol Evol. 2021 Dec 6;13(12):evab268. doi: 10.1093/gbe/evab268 (PMC8691055; doi:10.1093/gbe/evab268)
Supplement: evab268_Supplementary_Data [file evab268_supplementary_data.zip › Post_review_Supp_figuresS3.pdf]

Figure S3

A.

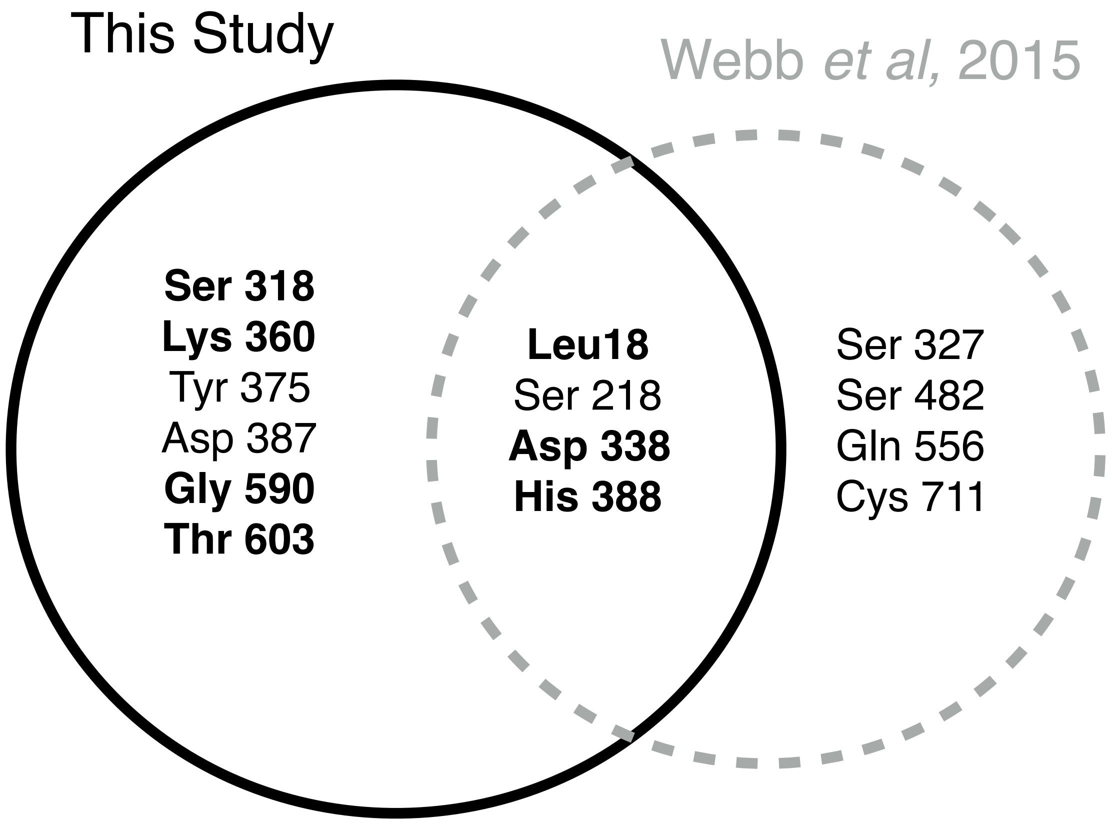

B.

mTRIF Site under Selection	P-value	Included in Experimental Analysis?
Leu 18	0.77	Yes
Ser 218	0.923	No – alternative codon used to encode serine in human TRIF protein. Selection at the level of codon choice potentially
Ser 318	0.945	Yes
Asp 338	0.610	Yes
Lys 360	0.912	Yes
Tyr 375	0.626	No – Human protein not aligned in this region
Asp 387	0.538	No – Very poor alignment in this region
His 388	0.605	Yes
Gly 590	0.745	Yes
Thr 603	0.714	Yes

Figure S3. A. Comparison of TRIF positive selection analysis results between current and previous study. Venn diagram showing the overlap of sites in TRIF identified as being under positive selection in mouse lineage. Residues highlighted **in bold** indicate those that whose function was experimentally tested in the context of the ancestral TRIF protein. **B.** Detailed accounting of TRIF sites under selection in mouse and reasons for exclusion in experimental analysis.
