# Supplementary material for: Adaptive Evolution in TRIF Leads to Discordance between Human and Mouse Innate Immune Signaling
Source: Genome Biol Evol. 2021 Dec 6;13(12):evab268. doi: 10.1093/gbe/evab268 (PMC8691055; doi:10.1093/gbe/evab268)
Supplement: evab268_Supplementary_Data [file evab268_supplementary_data.zip › Post_review_Supp_figuresS4.pdf]

Figure S4

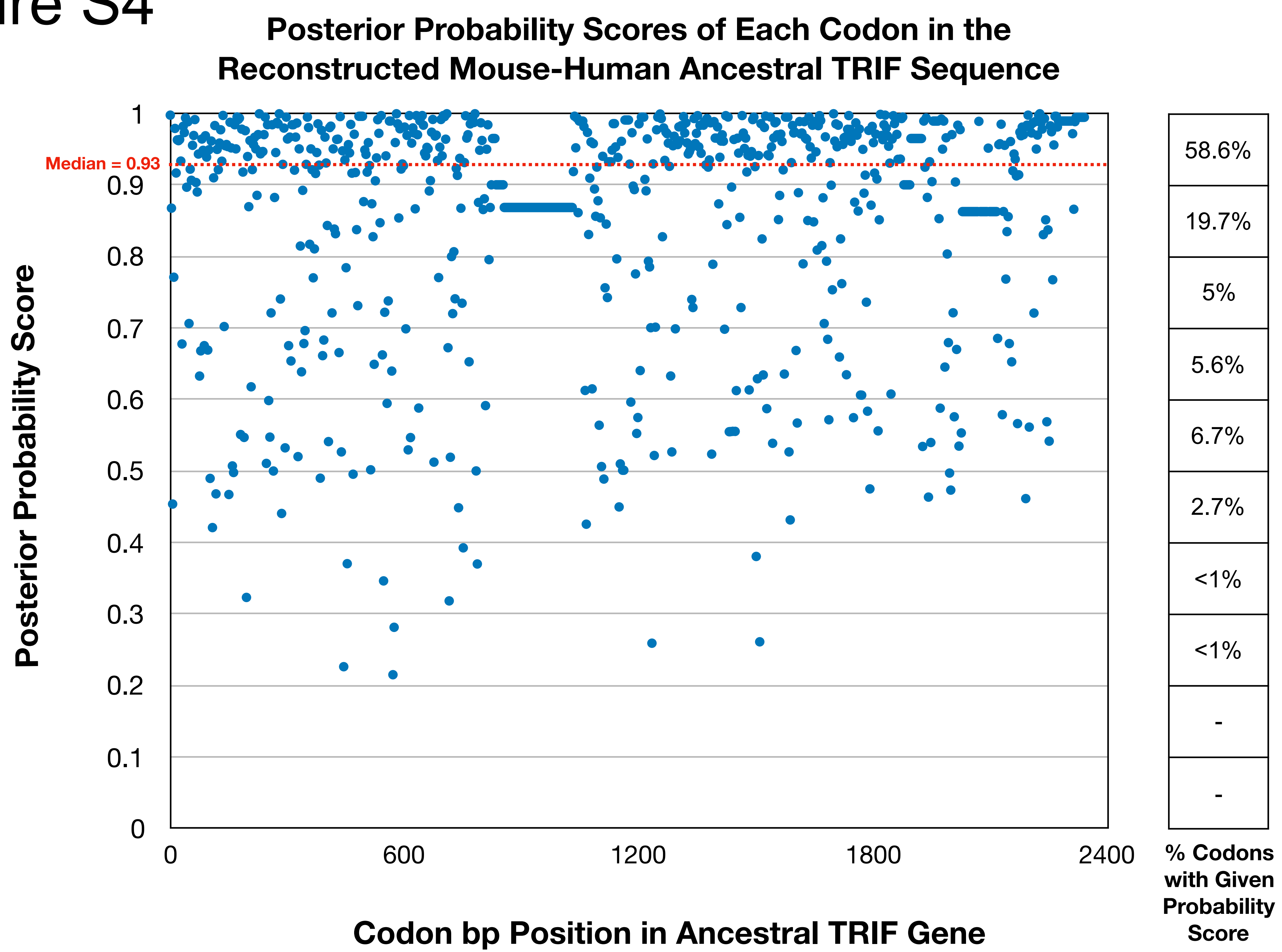

Figure S4. Statistical analysis of the predicted human-mouse ancestral TRIF (aTRIF) sequence. Each dot represents the posterior probability score for a single predicted codon along the length of the aTRIF gene. The median score is indicated at 0.93. Also indicated (on the right) is the % of aTRIF codons that fall within a given range of probability scores; for example, 58.6% predicted aTRIF codons have a probability score >0.9.
