# Supplementary material for: Adaptive Evolution in TRIF Leads to Discordance between Human and Mouse Innate Immune Signaling
Source: Genome Biol Evol. 2021 Dec 6;13(12):evab268. doi: 10.1093/gbe/evab268 (PMC8691055; doi:10.1093/gbe/evab268)
Supplement: evab268_Supplementary_Data [file evab268_supplementary_data.zip › Post_review_Supp_figuresS5.pdf]

Figure S5

A.

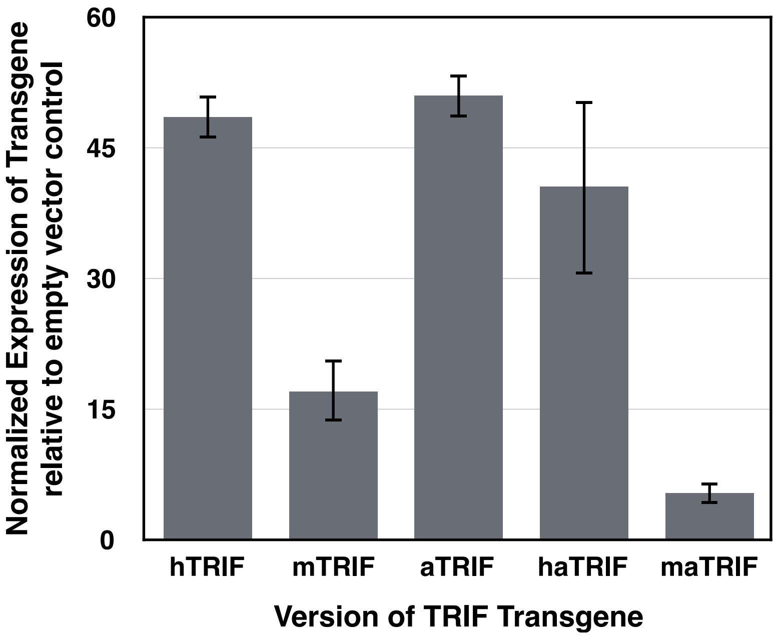

B.

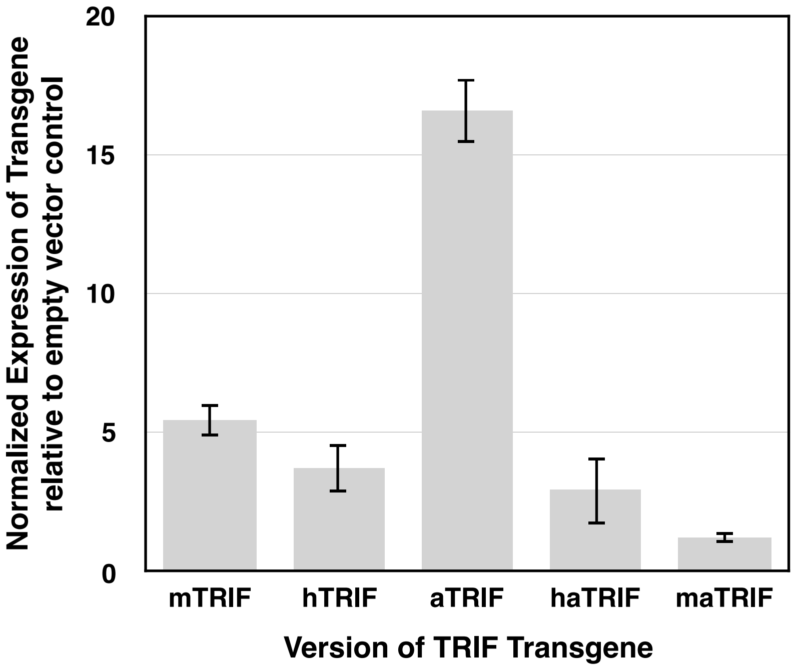

Figure S5. A. Quantitative RT-PCR analysis indicating the expression of each TRIF transgene in human HEK 293 TLR4 cells. and **B.** in murine 3T3 cells. For B and C, total RNA was extracted 12 hours post-transfection. TRIF gene expression was normalised to ACT1B and levels shown are relative to an empty vector control (set to 0). Data represent an average of 2 independent transfections, each assayed in triplicate.
