# Supplementary material for: Adaptive Evolution in TRIF Leads to Discordance between Human and Mouse Innate Immune Signaling
Source: Genome Biol Evol. 2021 Dec 6;13(12):evab268. doi: 10.1093/gbe/evab268 (PMC8691055; doi:10.1093/gbe/evab268)
Supplement: evab268_Supplementary_Data [file evab268_supplementary_data.zip › Post_review_Supp_figuresS6.pdf]

Figure S6

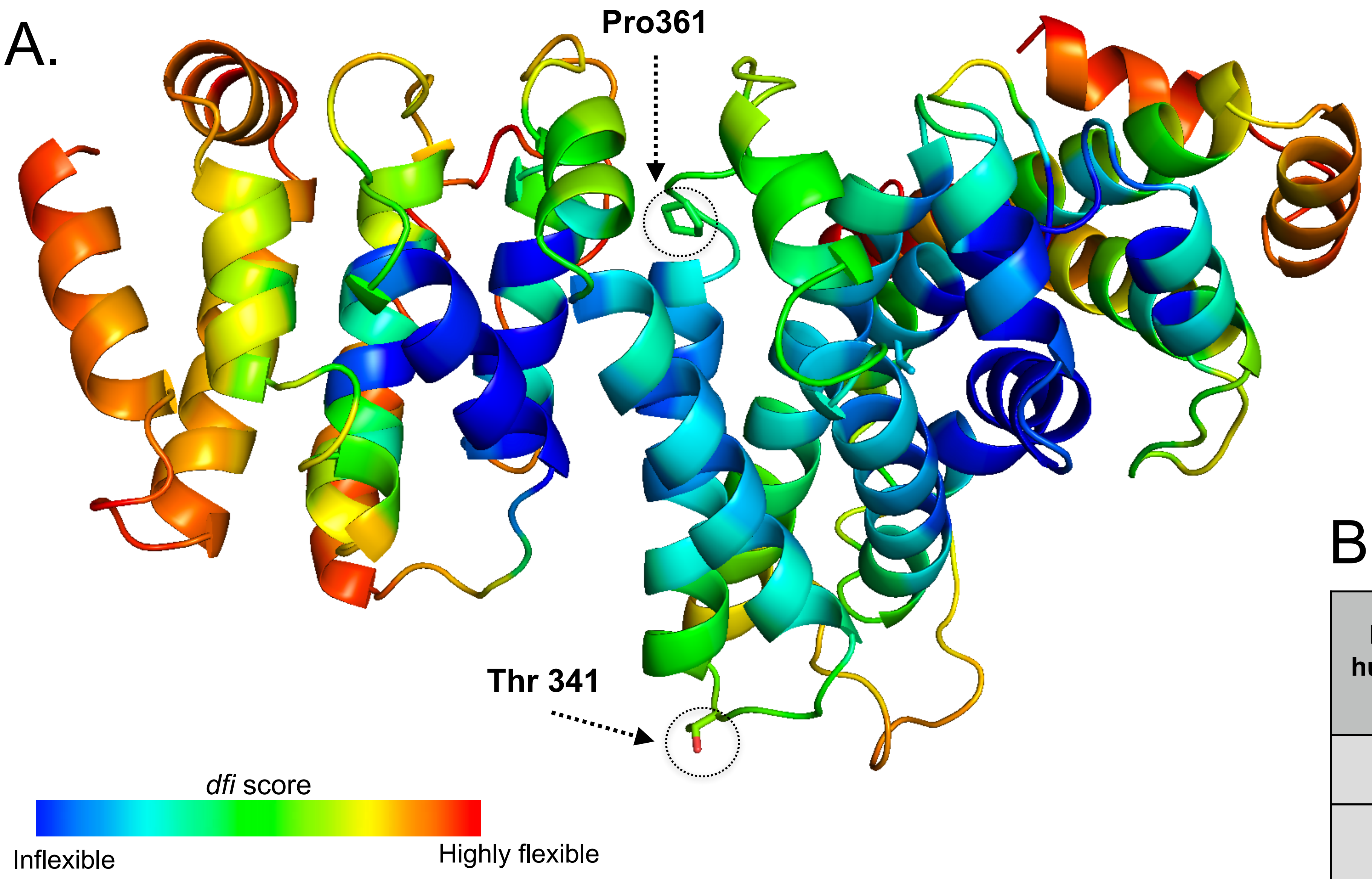

B.

Modelled human TRIF Residue	# Stabilising Mutations (ΔΔG < 1kcal/ mol)	# Destabilising Mutations (ΔΔG >1 kcal/ mol)
**Thr 341**	5	14
**Pro 361**	18	1

Figure S6. The predicted structure of a portion the human TRIF protein (residues Set 154 to Ala 605394) generated using MODELLER (Fiser and Sali 2003). Residues are highlighted based on their dynamic flexibility index, (*dfi)* score, as per the indicated scale, and the position of two human residues that confer function to the haTRIF protein are indicated. Structure representation was generated using pymol (Delano W.L. 2002). **C.** A summary of the *in silico* mutatgenesis analysis done on human TRIF protein structure using dezyme software (https://soft.dezyme.com). The number of mutations that led to either a decrease (stabilising) or increase (destabilising) in the folding free energy (△△G) of human TRIF is indicated.
