# Supplementary material for: Adaptive Evolution in TRIF Leads to Discordance between Human and Mouse Innate Immune Signaling
Source: Genome Biol Evol. 2021 Dec 6;13(12):evab268. doi: 10.1093/gbe/evab268 (PMC8691055; doi:10.1093/gbe/evab268)
Supplement: evab268_Supplementary_Data [file evab268_supplementary_data.zip › 210543_supplementary tables.docx]

**Table S1**: List of innate-immune response genes analysed using CodeML and the size of sequence dataset used for positive selection analysis.

| **Gene Name** | **Human Ensemble ID** | **Number of gene orthologues used for CodeML analysis (maximum = 43)** |
| --- | --- | --- |
| **TRIF** | ENSG00000243414 | 30 |
| **TLR3** | ENSG00000164342 | 29 |
| **IRF3** | ENSG00000126456 | 36 |
| **p38-β/MAPK11** | ENSG00000185386 | 26 |
| **JNK/MAPK8** | ENSG00000107643 | 39 |
| **TICAM 2** | ENSG00000243414 | 30 |
| **TNFα** | ENSG00000232810 | 37 |
| **IL-1B** | ENSG00000125538 | 40 |
| **IL-6** | ENSG00000136244 | 36 |
| **IL-12A** | ENSG00000168811 | 42 |
| **FOS** | ENSG00000170345 | 39 |
| **INF β1** | ENSG00000171855 | 28 |

**Table S2:** Excel file in supplementary information.

**Table S3**: **Amino acid sequence comparisons between ancestral TRIF (aTRIF), mouse TRIF(mTRIF) and human TRIF (hTRIF) proteins** at the three annotated TRIF domains, showing the number of residues in the alignment (# res.), % identity (%id), % similarity (%sim) and % difference (% diff).

|  | **TIR domain** | | | | **RHIM domain** | | | | **Traf6 binding domain** | | | |
| --- | --- | --- | --- | --- | --- | --- | --- | --- | --- | --- | --- | --- |
| **Proteins Aligned** | **# res.** | **% id** | **% sim** | **% diff** | **# res.** | **% id** | **% sim** | **% diff** | **# res.** | **% id** | **% sim** | **% diff** |
| **aTRIF, hTRIF, mTRIF** | 143 | 68.5 | 81.1 | 18.9 | 34 | 73.5 | 79.4 | 20.6 | 5 | 80.0 | 100.0 | 0.0 |
| **aTRIF, hTRIF** | 143 | 90.2 | 93.7 | 6.3 | 39 | 97.4 | 97.4 | 2.6 | 5 | 100.0 | 100.0 | 0.0 |
| **aTRIF, mTRIF** | 143 | 75.5 | 84.6 | 15.4 | 34 | 73.5 | 79.4 | 20.6 | 5 | 80.0 | 100.0 | 0.0 |

**Table S4**: **Primer sequences used in this study.**

| **Primer ID** | **Sequence (5’-3’)** | **Purpose** |
| --- | --- | --- |
| **oEMH1134** | CGTTACACTCCACCAACAGC | Mouse TRIF qRT-PCR forward primer |
| **oEMH1135** | GTTACATAGCTTGCTGGGCC | Mouse TRIF qRT-PCR reverse primer |
| **oEMH1132** | CCAAGCCATGATGAGCAACC | Human TRIF qRT-PCR forward primer |
| **oEMH1133** | AGATCTGGGAGTGTTCGTCC | Human TRIF qRT-PCR reverse primer |
| **oEMH1136** | CAGCAAGCTCTGTGATGACC | Ancestral TRIF qRT-PCR forward primer |
| **oEMH1137** | GGGCTAATTTCGTGGCTTGG | Ancestral TRIF qRT-PCR reverse primer |
| **oEMH1160** | AGTGCTGCCGTCATTTTCTG | Mouse IP-10 qRT-PCR forward primer |
| **oEMH1161** | TTCAAGCTTCCCTATGGCCC | Mouse IP-10 qRT-PCR reverse primer |
| **oEMH1164** | AGTGGCATTCAAGGAGTACC | Human IP-10 qRT-PCR forward primer |
| **oEMH1165** | AGCAATGATCTCAACACGTGG | Human IP-10 qRT-PCR reverse primer |
| **oEMH1144** | AGAAGATCTGGCACCACACC | Mouse ACTB qRT-PCR forward primer |
| **oEMH1145** | TCATCTTTTCACGGTTGGCC | Mouse ACTB qRT-PCR reverse primer |
| **oEMH1018** | CCAACCGCGAGAAGATGA | Human ACTB qRT-PCR forward primer |
| **oEMH1019** | TCCATCACGATGCCAGTG | Human ACTB qRT-PCR reverse primer |
